# Future scenarios for British biodiversity under climate and land-use change

**DOI:** 10.1038/s41467-026-70064-4

**Published:** 2026-03-31

**Authors:** Rob Cooke, Victoria J. Burton, Calum Brown, Colin A. Harrower, Steven M. White, Chris Huntingford, Rob Dunford-Brown, Richard Fox, Paula A. Harrison, Cang Hui, Dario Massimino, Andy Purvis, Emma L. Robinson, James Rodger, Nick J. B. Isaac, James M. Bullock

**Affiliations:** 1 UK Centre for Ecology & Hydrology, Maclean Building, Crowmarsh Gifford, Wallingford, UK; 2 Natural History Museum, London, UK; 3 Institute of Meteorology and Climate Research, Atmospheric Environmental Research (IMK-IFU), Karlsruhe Institute of Technology, Garmisch-Partenkirchen, Germany; 4 Highlands Rewilding Ltd., Inverness, UK; 5 Butterfly Conservation, East Lulworth, Wareham UK; 6 UK Centre for Ecology & Hydrology, Bailrigg, Lancaster UK; 7 Centre for Invasion Biology, Department of Mathematical Sciences, Stellenbosch University, Stellenbosch, South Africa; 8 British Trust for Ornithology, The Nunnery, Thetford, UK; 9 Georgina Mace Centre for the Living Planet, Imperial College London, Silwood Park, UK; 10 Department of Biological and Agricultural Sciences, Sol Plaatje University, Kimberley, South Africa

**Keywords:** Climate-change ecology, Community ecology, Ecological modelling, Socioeconomic scenarios, Biodiversity

## Abstract

Projections of biodiversity futures are needed to translate global policies into national action. We use dissimilarity modelling to project climate change scenarios for 1002 plant, 56 butterfly, and 219 bird species across Great Britain up to 2080. Under all scenarios we find extensive community reorganisation, with the disappearance of current bioclimates and emergence of novel ones. We also explore impacts of combined climate and land-use change, finding that even optimistic scenarios could see accumulating extinction debts. Scenarios featuring reduced emissions and a more sustainable society could bend the curve of loss, reducing species heading for extinction by 32% for plants, 14% for butterflies, and 20% for birds. Scenarios differ in impact between groups, with plants showing the most severe responses to environmental change. Overall, we show that actions taken during the next 20 years are crucial to mitigate the worst effects of climate and land-use change for biodiversity in Britain.

## Abstract (web summary)

Climate and land-use change are transforming biodiversity, yet national futures remain uncertain. The study projects growing extinction debts, but suggests that sustainable low-emission pathways can limit the worst impacts on British biodiversity.

## Introduction

Climate and land-use change are recognised as principal drivers of biodiversity change^1–3^. These threats are reorganising biodiversity, including species range shifts and compositional change^2,4^, as well as reducing species richness^5,6^. Thus, biodiversity is changing, with the disappearance of existing species and communities, and the emergence of novel communities^7^. Moreover, climate change and land-use change are both likely to accelerate in the future^1,8^, with increasing risks to biodiversity^9^, and to nature’s contributions to people^3,10^.

Although there is considerable evidence on how drivers have caused biodiversity decline in the past^1–3^, these drivers themselves are in flux. Thus, we cannot expect to bend the curve of biodiversity decline^9,11^ and meet the ambition of the Kunming–Montreal Global Biodiversity Framework (GBF)^12^ only by addressing the drivers of historic declines^8,13^. Anticipating future change in biodiversity and ecosystems is therefore one of the most pressing issues of contemporary ecology.

Biodiversity projections can improve our understanding of emerging and potential futures^14^, offering insights beyond the driving influences against which they are calibrated. Moreover, biodiversity projections enable decision-makers to adjust policies in line with potential future outcomes^15^. Yet, realistic biodiversity projections are challenging to produce, due to complex interconnections between society, the environment, and biological systems. Robust projections require all these features to be parameterised. To reduce the parameter space and capture major drivers of change, biodiversity scientists can use scenarios^16^. Scenarios describe coherent narratives of future socioeconomic and environmental conditions. Models then translate these scenarios into projected biodiversity outcomes based on quantitative driver-response relationships (e.g., climate change, land-use change)^17^. Hence, scenario modelling can be used to generate quantitative estimates of the future trajectories of biodiversity and explore plausible pathways to desirable futures.

Scenario modelling has been used effectively in climate change research to inform climate mitigation and adaptation, and to identify impactful suites of actions^18^. For instance, Representative Concentration Pathways (RCPs) describe a spectrum of climate scenarios based on greenhouse gas emissions^19^. In parallel, Shared Socio-economic Pathways (SSPs) have been developed to describe plausible future trajectories of societal development^20^, capturing multiple aspects of social structures, economics, and resource use. By combining SSPs with RCPs, we can project biodiversity responses under a range of plausible scenarios, constrained by a coherent and internally consistent set of assumptions about key driving forces and relationships. Scenarios that include both climate and biodiversity have been explicitly recommended as a research priority^21^ and provide an opportunity for productive collaborations between the climate and biodiversity communities^22^. Moreover, an improved capacity to project biodiversity changes and their responses to divergent future scenarios and mitigation strategies has been highlighted as a research need^1,14^.

There have been several studies of global biodiversity scenarios in recent years (e.g., refs. ^9,23^). Although informative, these global analyses alone are insufficient. Indeed, our repeated failure to achieve agreed global biodiversity targets may be inevitable given the lack of appropriate implementation and effective actions toward them at national and subnational levels^24^. Thus, to inform conservation action, there is a need for national, spatially-explicit biodiversity projections^25^, to provide the specific, detailed, and contextual information that is not present within global analyses.

Here, we explore the magnitude and spatial distribution of biodiversity change in Great Britain (hereafter Britain) under a range of climate and land-use scenarios up to the year 2080. We use an innovative approach based on compositional dissimilarity modelling^23,26^ to consider cross-taxon responses rather than single species. We go beyond and enrich previous global analyses of biodiversity futures by using high-resolution, co-developed scenarios for plausible British climates^27^, land use^28^, and socio-economic conditions^29^, generating fine-scale measures of biodiversity across three taxonomic groups. We project four metrics of biodiversity change for Britain. These biodiversity metrics reflect different attributes of biodiversity and ecological processes, including the loss of natural environments, the emergence of novel environments, and the regional extinction of species. These metrics therefore, reflect key components of the GBF^12^.

**Fig. 1:**
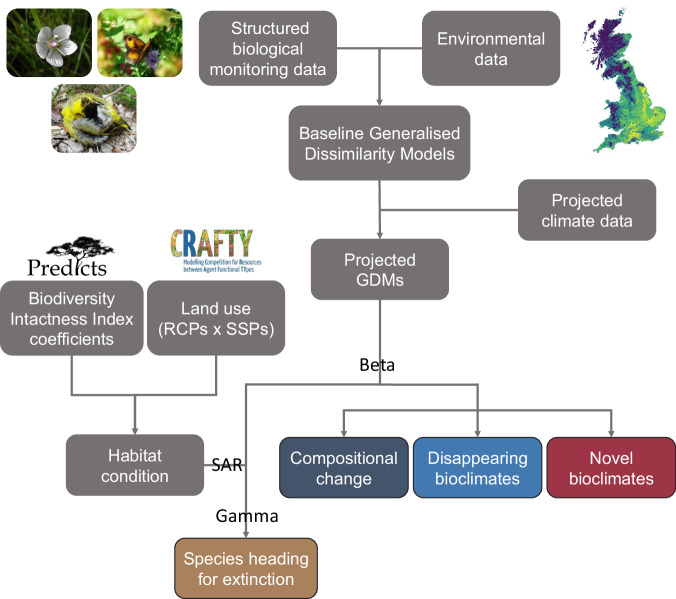
Methodological overview. Structured biological monitoring data (1002 plant, 56 butterfly, and 219 bird species) is coupled with contemporary environmental data in generalised dissimilarity models (GDMs) for the baseline period (1980–2020). These baseline GDMs are then projected up to 2080 based on projected climate changes (Representative Concentration Pathways; RCPs). From these projections multiple beta diversity-based metrics are calculated: compositional change, disappearing bioclimates, and novel bioclimates. Finally, using the species-area relationship (SAR), a proportional loss of habitat, informed by projections of future habitat condition and the projected GDMs, is translated into the number of currently native species heading for extinction from Great Britain. See “Methods” for further details of each step. SSPs Shared Socioeconomic Pathways. Photographs are by Rob Cooke (plant—Grass-of-Parnassus *Parnassia palustris*; butterfly—Gatekeeper *Pyronia tithonus*; bird—Eurasian Siskin *Spinus spinus*). The PREDICTS and CRAFTY logos were created by the respective project teams and are reproduced with permission.

We start by coupling structured biological monitoring data with high-resolution (1 km) environment variables using generalised dissimilarity models (GDMs; Fig. 1). Specifically, we include three static environmental variables (two topographic variables and one soil). As well as seven climate variables that change over time, covering aspects of temperature, precipitation, evapotranspiration, and sunshine (see “Methods” for more details). Hence, we use GDMs to relate compositional dissimilarity between pairs of sites (i.e., beta diversity) to differences in the environment^26^, providing a framework for projecting community responses through time to climate change. GDMs have advantages over other common approaches, such as species distribution models (SDMs), by considering communities of species in combination, and therefore implicitly including species interactions. They also require fewer parameters to model community responses and therefore allow rare species to be included^26^.

We parameterise our GDMs for the present day using structured monitoring data for 1002 plant species (~72% of British native plant species; National Plant Monitoring Scheme^30^), 56 butterfly species (~97% of British native butterfly species; UK Butterfly Monitoring Scheme (UKBMS)^31^), and 219 breeding bird species (~96% of British native breeding bird species; UK Breeding Bird Survey^32^), representing a broad range of functional/trophic groups.

We then project these GDMs to 2020–2040 (centred on 2030, hereafter referred to as 2030), 2040–2060 (hereafter 2050), and 2060–2080 (hereafter 2070) under climate change scenarios, based on RCPs specific to Britain that are consistent with global projections^27^ (Supplementary Tables 1 and 2). Our framework implements the simplifying assumption that changes in beta diversity (variation in species composition across communities within a region) are primarily driven by climate change^2,4^, while changes in gamma diversity (total species richness in a region) are driven by both climate and land-use change^5,6^ (Fig. 1). Thus, we start by quantifying the impact of future climate change on beta diversity, using multiple metrics to capture different ecological processes (Supplementary Fig. 1): compositional change—the magnitude of compositional dissimilarity between the present and future; disappearing bioclimates—current species-climate combinations with no future analogue; and novel bioclimates—future species-climate combinations with no current analogue^26^.

We then quantify the impact of climate and land-use change on gamma diversity by calculating the number of species heading for extinction (sometimes referred to as committed to extinction^23,33^). Here, species heading for extinction are native species expected to disappear from their entire British range under the driver values for the future scenario. Together, these species form an extinction debt—the number of extant species that are expected to eventually become extinct due to adverse environmental changes^34,35^. Extinction debts require years to centuries to pay, depending on species’ current abundance, range size, and life-history traits^34,35^. To calculate species heading for extinction, we translate a proportional loss of habitat, driven by climate and land-use change, into an expected loss of species using the species-area relationship^23^. We calculate species heading for extinction under six plausible futures (i.e., scenarios constrained by realistic and credible trends based on current knowledge)—built from coherent combinations of RCPs and SSPs (Supplementary Table 1). We use five SSPs downscaled and enriched for the UK^29^ but embedded within the global^20^ and European SSPs^36^: SSP1 (sustainability), SSP2 (middle of the road), SSP3 (regional rivalry), SSP4 (inequality) and SSP5 (fossil fuelled development). We combine these with four RCPs (2.6, 4.5, 6.0 and 8.5) to provide six scenarios: RCP2.6-SSP1, RCP4.5-SSP2, RCP4.5-SSP4, RCP6.0-SSP3, RCP8.5-SSP2, and RCP8.5-SSP5 (see Supplementary Table 2 for more detail). These scenarios cover lower-end (RCP2.6) to higher-end (RCP8.5) climate change, as well as future societies with high and low challenges to adaptation and mitigation (Supplementary Table 2). Together, these scenarios cover a broad range of possibilities, contrasting different RCPs with the same SSP (RCPs 4.5 and 8.5 with SSP2), and different SSPs with the same RCP (SSPs 2 and 4 with RCP4.5; SSPs 2 and 5 with RCP8.5) (Supplementary Table 1).

## Results

### Compositional change—compositional dissimilarity between the present and future due to climate change

**Fig. 2:**
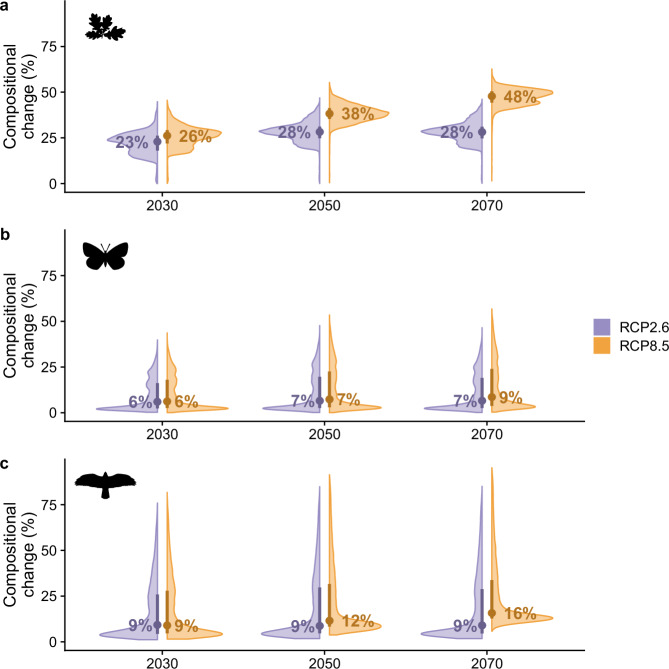
Compositional change for plants, butterflies, and birds in response to climate change to the year 2070 for Great Britain. Compositional change (compositional dissimilarity between the present and future) is shown for (**a**) plants (1002 species), (**b**) butterflies (56 species), and (**c**) breeding birds (219 species) under Representative Concentration Pathways (RCP) 2.6 (purple) and 8.5 (orange), extremes of low and high emission scenarios, for the years 2020–2040 (centred on 2030), 2040–2060 (centred on 2050), and 2060–2080 (centred on 2070). Points reflect median compositional change (also annotated), bars characterise the interquartile range, and the envelopes reflect the distribution of compositional change across all grid cells per scenario and taxonomic group. For full spatial results, see Supplementary Figs. 2–4. The icons are all from PhyloPic.org under Public Domain Dedication 1.0 licences (see collection https://www.phylopic.org/collections/5c4e4eea-1b95-408a-949d-d29f1e24a0fa). Icons are by Ferran Sayol (plant), Mattia Menchetti (butterfly), and Andy Wilson (bird).

Our first analysis quantifies the impact of future climate change (RCPs only) on beta diversity, with climate change a major driver of compositional change^2,4^. We identify notable climate change-driven shifts in composition across all three taxonomic groups, even under mild climate scenarios (Supplementary Figs. 2–4). For plants we project a median compositional change (the magnitude of compositional dissimilarity between the present and future; Supplementary Fig. 1) of 28% under RCP2.6 by 2070, and 48% under RCP8.5 (Fig. 2a). In other words, for a community of, say, 100 plant species under RCP8.5, 48 would not be shared between the present and 2070. We project a lesser, but still notable, change for butterflies of 6% under RCP2.6 by 2070 and 9% under RCP8.5, while for birds we project 9% under RCP2.6 and 16% under RCP8.5 (Fig. 2b, c). Thus, compositional change is at least 1.5 times greater under RCP8.5 compared to RCP2.6 (plants = 1.7 times greater, butterflies = 1.5, birds = 1.8; Fig. 2). Spatially, plants show dissimilar patterns to both butterflies (Spearman’s rank correlation *ρ* = −0.30 under RCP8.5 for 2070; Supplementary Figs. 2 and 3) and birds (*ρ* = −0.16; Supplementary Figs. 2 and 4), while butterflies and birds show similar patterns (*ρ* = 0.83; Supplementary Figs. 3 and 4). Overall, under all climate scenarios, we project ecologically significant changes in species composition across Britain.

### Changes to current bioclimates

Bioclimates are the integration of biological (species composition) and climatic (see “Methods” for details on three temperature variables, two precipitation variables, evapotranspiration variable and sunshine variable) components (Fig. 1). That is, rather than describing a location solely by its climate^37^, we describe a location by its climatic ability to support predicted species communities. In this context, we define analogues as current bioclimates that are <10% dissimilar (i.e., >90% similar) to any future bioclimate. We selected a dissimilarity threshold of 10% as it reflected a balance where not every pair of bioclimates was identified as different, yet it remains sensitive enough to identify analogue bioclimates^38^. Disappearing bioclimates are then current bioclimates that are ≥10% dissimilar (≤90% similar) to any future bioclimate (Supplementary Fig. 1). Essentially, disappearing bioclimates are current bioclimates that do not closely match future bioclimates. By contrast, novel bioclimates are future bioclimates that are ≥10% dissimilar (≤90% similar) to any current bioclimate (Supplementary Fig. 1). Thus, novel bioclimates capture the emergence of species-climate combinations without present-day equivalents.

### The disappearance of current bioclimates

**Fig. 3:**
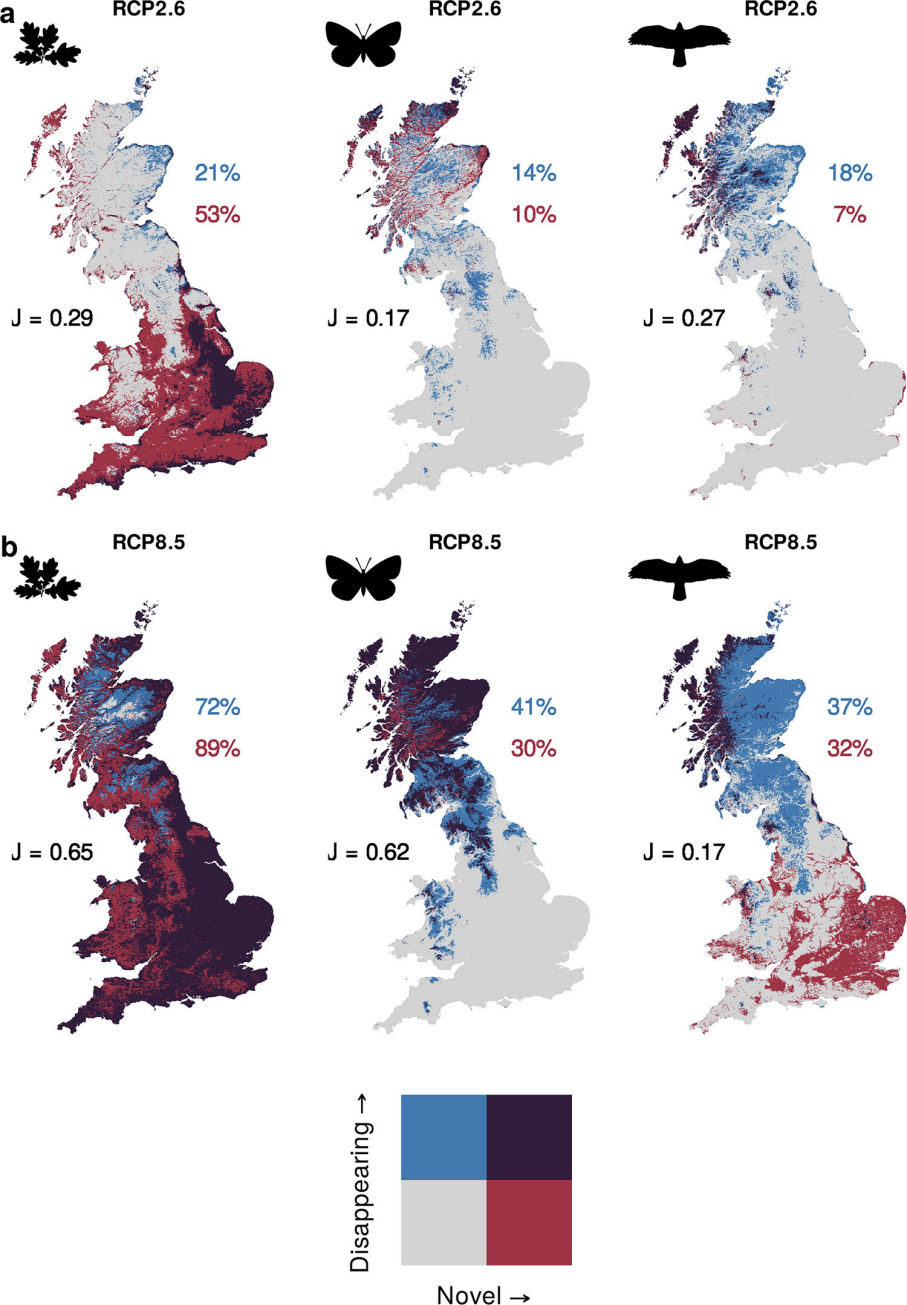
The co-location of disappearing and novel bioclimates for plants, butterflies, and birds across Great Britain for 2070. Disappearing bioclimates (≥10% dissimilarity between a focal current bioclimate and all future bioclimates across Britain—looking forward) and novel bioclimates (≥10% dissimilarity between all current bioclimates across Britain and a focal future bioclimate—looking backward) are shown for plants (1002 species), butterflies (56 species), and breeding birds (219 species). The projections for all panels are for 2060–2080 (centred on 2070; the final time slice and thus where patterns are most apparent) under Representative Concentration Pathways (RCP) (**a**) 2.6 and (**b**) 8.5 (extremes of low and high emission scenarios). The percentages are the area of Britain covered by disappearing bioclimates (blue) or novel bioclimates (red). The Jaccard similarity index (J = intersection/union; J = purple/(blue + red + purple)) indicates the spatial overlap compared to the combined area of disappearing and novel bioclimates. For full results across time slices and RCPs for disappearing bioclimates see Supplementary Figs. 5–7 and for novel bioclimates Supplementary Figs. 8–10.

We project the disappearance of current bioclimates across many locations (Fig. 3). Indeed, if anthropogenic emissions follow RCP2.6, disappearing plant bioclimates will be found across 21% of Britain (Fig. 3a). This increases to 37% if emissions follow RCP4.5 (Supplementary Fig. 5), 40% for RCP6.0 (Supplementary Fig. 5), and 72% if emissions follow RCP8.5 (3.4 times greater than RCP2.6; Fig. 3b). Butterflies show smaller areas of disappearing bioclimates, with 41% of Britain having disappearing bioclimates for RCP8.5 (2.9 times greater than RCP2.6), with these found especially in northern/upland areas (Fig. 3b). Birds show relatively similar spatial patterns to butterflies for disappearing bioclimates (Fig. 3), which cover 37% of Britain under RCP8.5 (2.1 times greater than RCP2.6).

The most dissimilar disappearing plant bioclimates (i.e., using a continuous measure of disappearing bioclimates) under RCP8.5 for 2070 are found along the southern and eastern coasts of Britain, as well as across large river catchment areas in eastern England (Supplementary Fig. 5). Whilst for butterflies and birds under RCP8.5 for 2070 (similar patterns between butterflies and birds; *ρ* = 0.78), the most dissimilar disappearing bioclimates are found across British uplands (Supplementary Figs. 6 and 7); suggesting that upland specialist species may be lost as these bioclimates disappear under future climate scenarios (Fig. 3).

### The emergence of novel bioclimates

We also see the emergence of novel bioclimates (Fig. 3). We see widespread novel bioclimates for plants under RCP2.6—53% of Britain (Fig. 3a), particularly across southern England, rising to 66% under RCP4.5, 68% under RCP6.0 (Supplementary Fig. 8), and 89% under RCP8.5 (1.7 times greater than under RCP2.6; Fig. 3b). Novel bioclimates for butterflies and birds are less widespread. For butterflies, novel bioclimates cover 10% of Britain under RCP2.6 (Fig. 3a), and 30% under RCP8.5 (3.0 times greater than under RCP2.6; Fig. 3b). For birds, novel bioclimates cover 7% of Britain under RCP2.6 (Fig. 3a), and 32% under RCP8.5 (4.6 times greater than under RCP2.6; Fig. 3b). Although the area coverage of novel bioclimates is similar for butterflies and birds, the spatial distribution of novel bioclimates differs between these taxa (Supplementary Figs. 9 and 10).

We project the most dissimilar novel bioclimates for plants under RCP8.5 for 2070 across central and eastern England (Supplementary Fig. 8). By contrast, the most dissimilar novel bioclimates for butterflies are spread across Scotland (Supplementary Fig. 9). For birds, we see novel hotspots across western Scotland, and the Lake District and Eryri (Snowdonia) National Parks (Supplementary Fig. 10), contrasting to patterns for butterflies (*ρ* = −0.30 RCP8.5 for 2070).

### Co-location of disappearing and novel bioclimates

We find that under RCP2.6 disappearing and novel bioclimates are generally not co-located (all Jaccard similarity indices <0.3; Fig. 3a). Many locations with disappearing bioclimates will exist under a future bioclimate that is found elsewhere today (i.e., these disappearing bioclimates are replaced by other current bioclimates; blue cells Fig. 3a), and many grid cells that are predicted to support novel bioclimates, currently operate under a bioclimate that is found elsewhere in the future (red cells Fig. 3a).

However, under more intense climate change, RCP8.5, we find greater overlap between disappearing and novel bioclimates for plants (*J* = 0.65) and butterflies (*J* = 0.62), but not for birds (*J* = 0.17). We identify a high conditional probability under RCP8.5 for plants (88%) and butterflies (67%) of disappearing bioclimates being replaced by novel bioclimates (purple cells Fig. 3b). In other words, plant and butterfly disappearing bioclimates are likely to be replaced by novel bioclimates, while for birds disappearing bioclimates are likely to be replaced by other current bioclimates, with novel bioclimates emerging in new locations.

### Species heading for extinction

Finally, we estimate the number of species heading for extinction (native species projected to disappear from their entire British range over the long term). We quantify species heading for extinction under six plausible futures (combinations of RCPs, climate, and SSPs, socioeconomic factors). These scenarios therefore capture the major driving forces of climate change and land-use change, which play key roles in driving species regional and global extinctions^3,39,40^.

It is worth noting that there is already an extinction debt for Britain (Supplementary Fig. 11), reflecting the fact that some species are heading for extinction due to past changes in climate and land use^41^. Moreover, 54 plant species, four butterfly species, and nine breeding bird species have gone extinct since 1500 CE, primarily since 1900 CE^42^.

**Fig. 4:**
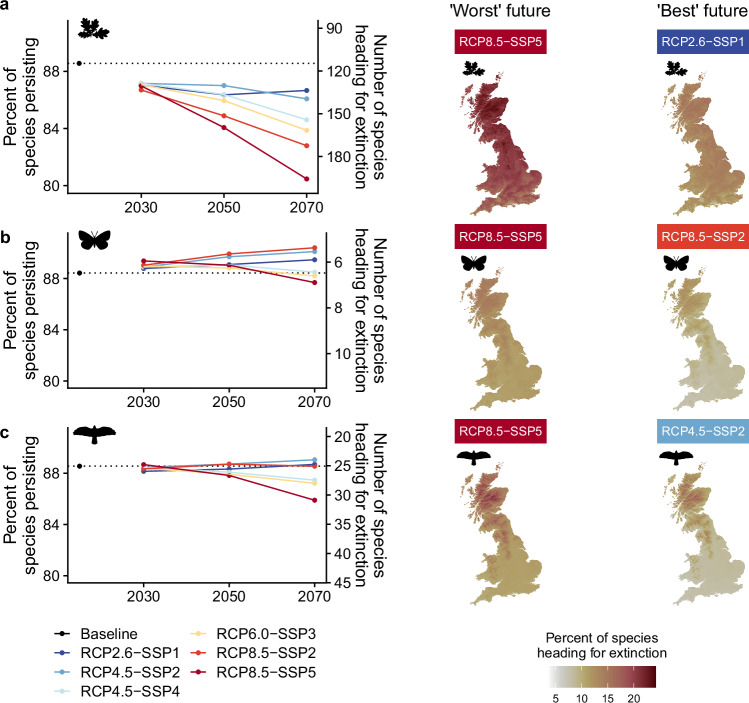
Projected trends and spatial patterns of species heading for extinction, in response to climate change and land-use change, to the year 2070 for Great Britain. The percent of species persisting and its converse, species heading for extinction, are plotted for six plausible futures—combinations of Representative Concentration Pathways (RCP) and Shared Socioeconomic Pathways (SSP) (Supplementary Tables 1 and 2). **a** Plants (1002 species), (**b**) butterflies (56 species), and (**c**) breeding birds (219 species). The baseline is set to 2015, reflecting the baseline land use map, but more generally applies to the period 1980–2020 to which the scenarios are anchored (see Supplementary Fig. 11 for subtle differences between baselines for the different taxonomic groups). We also show maps for the worst (greatest number of species heading for extinction) and best (fewest species heading for extinction) futures in 2070 for each taxonomic group. All projections represent combined land-use and climate change effects. For full spatial results across time slices and scenarios, see Supplementary Figs. 12–14.

We see diverging future responses between the different taxonomic groups (Fig. 4). For plants, a future of low global emissions and a more sustainable societal pathway for Britain, RCP2.6-SSP1, can bend the curve of species extinctions (Fig. 4a). Indeed, under RCP2.6-SSP1, the number of species heading for extinction across Britain worsens from 115 (of 1002 plant species; 11%) for the baseline period to 130 (13%) in 2030, 137 (14%) in 2050, and then improves to 134 (13%) in 2070. So, even under the best scenario, we project a substantial extinction debt, equivalent to 2.5 times the number of historic extinctions. Under more climatically severe and less sustainable societal scenarios, we see the rapid and accelerating accumulation of extinction debts. For instance, under RCP8.5-SSP5, 196 plant species (20% of plant species; 3.6 times the number of historic extinctions) will be heading for extinction by 2070 (some of these extinctions will occur after 2070), 1.5 times more plant species than under the best future for plant biodiversity (RCP2.6-SSP1).

Butterflies and birds generally do not decline under any scenario in the short term (until 2050; Fig. 4b, c)—although, this is in comparison to an already highly degraded baseline (i.e., many species are already heading for extinction)^41^. However, after 2050 the projections diverge, with the worst trajectories under RCP8.5-SSP5 (by 2070, seven butterfly species are heading for extinction, 12% of butterflies; 31 bird species, 14% of birds), followed by RCP6.0-SSP3 (seven butterflies, 12%; 28 birds, 13%) and RCP4.5-SSP4 (six butterflies, 11%; 27 birds, 13%). Whereas under the other three scenarios butterflies and birds show more positive responses, including under SSP2 regardless of the magnitude of climate change (RCP8.5 and RCP4.5) and under RCP2.6-SSP1 (Fig. 4b, c). Although we project a small absolute number of butterflies heading for extinction (5–7 species), this is over 10% of Britain’s current butterfly fauna and these extinctions would be 1.3–1.8 times the number of historic extinctions (four historic extinctions over the past 160 years). For birds, under the worst future we project a substantial extinction debt, equivalent to 3.4 times the number of historic extinctions (nine historic breeding bird extinctions).

Comparing RCP4.5 and RCP8.5 under the same SSP (SSP2), we find a greater difference for plants (140 species heading for extinction under RCP4.5-SSP2 vs 172 under RCP8.5-SSP2 by 2070; Fig. 4a), compared to butterflies (six vs five; Fig. 4b) or birds (24 vs 25; Fig. 4c)—which may imply that butterflies and birds are potentially less sensitive to climate change driven extinctions than plants. Furthermore, we find that variation in socio-economic factors (i.e., SSPs) drive large differences in the number of butterflies and birds heading for extinction, sometimes dominating the effects of climate change. For instance, the differences between RCP8.5-SSP2 and RCP8.5-SSP5 (five species heading for extinction under RCP8.5-SSP2 by 2070 vs seven under RCP8.5-SSP5 for butterflies; 25 vs 31 for birds; Fig. 4b, c), are greater than the differences between RCP4.5-SSP2 and RCP8.5-SSP2 (see above).

Overall, we find that differences between scenarios accelerate into the future across taxa, especially over longer timescales (e.g., by 2070). Hence, we find that by 2070, mitigation consistent with the Paris Agreement (RCP2.6-SSP1) would reduce the number of species heading for extinction by 32% for plants, 14% for butterflies, and 20% for birds compared to RCP8.5-SSP5. Furthermore, we find that the GBF’s global goal to reduce extinction rate and risk tenfold by 2050^12^ would be missed in Britain for plants, butterflies, and birds under all scenarios, even the least severe (RCP2.6-SSP1). Moreover, England’s target to reduce the risk of species’ extinction by 2042 compared to 2022^43^, when converted to a baseline-2050 comparison, would be missed for plants for all scenarios, would be met for butterflies for all scenarios, and for birds would be met for two scenarios (RCP4.5-SSP2 and RCP8.5-SSP2) but missed for four scenarios (RCP2.6-SSP1, RCP4.5-SSP4, RCP6.0-SSP3, and RCP8.5-SSP5).

## Discussion

Using an innovative modelling approach focussing on community-scale responses, we project both local and national biodiversity change for multiple taxonomic groups under alternative scenarios using best-available datasets. Our approach offers the advantage of estimating not only the local impact of environmental change, but also how this scales up to the collective biodiversity of a region. Indeed, our projections are not simply expressing absolute climate change or land-use change but reflect how biological communities respond to change. We therefore make a step towards nationally and globally applicable models describing biodiversity interactions with climate and land-use change^22^. We provide a systematic quantification of potential future climate-induced biodiversity change, as well as climate and land-use driven extinction debt. Overall, we find extensive compositional change, the disappearance of current bioclimates and the emergence of novel bioclimates, as well as the accumulation of species extinction debts.

Considering climate change alone, the high-end climate change scenario, RCP8.5, represents a high fossil fuel development world and a reversal of climate policies throughout the 21st century^44^. Under this scenario, our analysis predicts that Britain will experience widespread species reorganisation by 2070, especially for plants (48%), and at lower levels for butterflies (9%) and birds (16%). While current trajectories look to be more closely tracking RCP6.0 and RCP4.5^45,46^, under these scenarios, we still see pervasive compositional change for plants (35% for RCP6.0, 34% for RCP4.5), butterflies (7% for both), and birds (11% for both). These levels of biodiversity change are inherently risky^47^. Indeed, compositional change can lead to further unmodelled repercussions, including the rewiring of food webs and networks^48^, cascading extinctions^39^, ecological surprises^49^ and altered rates of ecosystem processes and functions^50^, compromising nature’s contributions to people^10^.

The extensive species reorganisation we identify leads to the disappearance of current bioclimates for plants (72% of Britain under RCP8.5), butterflies (41%) and birds (37%). Thus, we project the loss of existing bioclimates, along with their associated biological and climatic configurations, structures, and functions^48,51^. These processes move systems away from Goal A of the GBF, which requires that the integrity, connectivity and resilience of all ecosystems are maintained, enhanced, or restored, substantially increasing the area of natural ecosystems by 2050^12^.

We also identify the emergence of novel bioclimates for plants across almost all of Britain (89%), and across almost one third of Britain for butterflies (30%) and birds (32%). Thus, we find that future climate change could push bioclimates to novel structures over human timescales. The high prevalence of novel bioclimates reflects the magnitude and speed of current and future climate change, combined with the transient nature of current ecological communities as we know them. Still, for these novel bioclimates to be realised, species would need to colonise new areas sufficiently quickly, which is known to be severely limited by species’ dispersal abilities and the low permeability of anthropogenic landscapes^52,53^. Protected area networks are likely to be essential to help species survive and track suitable bioclimatic spaces^54–56^. However, these novel bioclimates are expected to be biologically poorer than projected and biased towards successful dispersers and colonisers, with colonisation credits that may take centuries to be realised^35^. Even where our projections indicate the redistribution of current bioclimates, these areas may develop *de facto* novel structures and interactions due to delays in species establishment.

Our analytical approach makes the simplifying assumption that changes in beta diversity are primarily driven by climate change, while changes in gamma diversity are driven by both climate change and land-use change. These alternative dimensions of biodiversity reveal different insights about biodiversity change. Where we have interwoven climate and land-use change, we can quantify their combined impact on species extinction (changes in gamma diversity). Specifically, we find that under the most extreme scenario in terms of climate change and unsustainable societal change, RCP8.5-SSP5, by 2070, 196 current native plant species (20% of plant species), seven butterfly species (12%) and 31 bird species (14%) are predicted to be driven towards extinction in Britain. This leads to regional extinction debts that are greater than historical levels for plants (3.6 times the number of historic extinctions), butterflies (1.8), and birds (3.4), as well as missing global^12^ and national^43^ species extinction targets under the majority of scenarios for plants and birds. Moreover, species not included in the analysis (~28% of British native plant species; ~3% of butterfly species; ~4% of breeding bird species) lacked data and are likely rare, and therefore intrinsically more likely to go extinct. Hence, these estimates are likely conservative.

We find that RCP8.5-SSP5 is universally the worst plausible future for biodiversity. The major characteristics of RCP8.5-SSP5 (see Supplementary Table 2 for brief descriptions and key features of plausible futures) are high-end climate change combined with very high levels of agricultural intensification, high demands for livestock products (and associated agricultural conversion), widespread urban expansion, increased pollution, and weakened regulations to protect the environment^28,29^. There is clear evidence linking these features to negative impacts on biodiversity^1^. By contrast, there is no universally best plausible future for biodiversity. For plants, RCP2.6-SSP1 is the best future, even bending the curve of biodiversity decline^9,11^ in the later stages of the scenario, reflecting the underlying storyline^29^. RCP2.6-SSP1 is consistently among the best futures for all taxa, along with RCP4.5-SSP2, which is the best future for birds, even though the outcomes for biodiversity are not overwhelmingly positive. These two futures share some features (Supplementary Table 2), including an increase in mixed woodland and a decrease in intensive pasture area due to a drop in meat and milk demand. Even though the wider consequences are often assumed to be more beneficial under SSP1 narratives, they depend on optimistic assumptions of the adoption of agricultural practices capable of achieving yield growth alongside deintensification^57^, while SSP2 narratives depend on policies that couple yield increases with habitat restoration on spared land, which might not occur^58^. Thus, more ambitious and positive scenario frameworks for biodiversity are needed, such as the recently developed Nature Futures Framework that embraces a plurality of perspectives on desirable futures for nature and people^59^ to provide integrated evidence on what actions work for biodiversity.

Overall, we find that high-end climate change (RCP8.5 and RCP6.0) amplifies biodiversity change. Such changes include increased compositional change, more widespread disappearing and novel bioclimates, and a greater extinction debt. As a minimum, we find that by 2070, mitigation consistent with the Paris Agreement (RCP2.6-SSP1) would reduce species heading for extinction by 32% for plants, 14% for butterflies, and 20% for birds compared to RCP8.5-SSP5. Yet national pledges of emissions reductions under the Paris Agreement fall well short of those required to meet the agreed targets, with modelling studies estimating a median warming of 2.6–3.1 °C—more closely aligned with RCP4.5 and RCP6.0^60^. Our findings reinforce the need for stronger international commitments to emissions reductions, echoing earlier calls informed by historic climate-driven changes in British biodiversity^61^. Although our findings are regional, they highlight that halting future biodiversity loss in Britain will ultimately depend on international coordinated action to reduce total emissions.

We are cautious about making direct comparisons between our findings and previous studies, as our scenarios are intentionally explorative and differ in structure (Supplementary Table 2)^27–29^ from those used in other analyses of British biodiversity. Nonetheless, our results broadly align with previous projections of climate change impacts on British biodiversity. Specifically, they correspond with recurring patterns reported across multiple studies: widespread compositional change across Britain^62,63^; high sensitivity of upland communities to climate change^62–67^; loss of suitable climate space in southern England and East Anglia for plants^64,68,69^; and particularly severe impacts for plants^65^. While these patterns are consistent across studies, including ours, the magnitude and spatial distribution of changes vary, reflecting differences in methodology, taxonomic focus, and climate scenarios.

At present, our model is an equilibrium time slice description linking biodiversity and climate. A future advance would be to introduce transient effects into the model that explicitly account for the time lags in biodiversity changes^70^. This would help answer whether biodiversity levels can remain high for more substantial amounts of climate change, but conditional on reaching those levels of climate change more slowly. These advances would likely require more mechanistic or process-based approaches^71,72^, although these approaches can be challenging to apply at large taxonomic and/or geographic scales. Still, we reveal temporal patterns of biodiversity change. Specifically, we find that the divergence between unsustainable futures (RCP8.5-SSP5) and more sustainable futures (RCP2.6-SSP1 and RCP4.5-SSP2) becomes more apparent from the 2050 s time slice onwards^73^. Thus, we show that actions during the next 20 years are critical to mitigate the worst effects of climate and land-use change for biodiversity in Britain.

In combination with committed action on climate, our analysis highlights the need to make positive land use decisions for nature. Indeed, there is increasing recognition that meeting UK policy goals for climate change and biodiversity are unlikely to be met without fundamental land reform^74,75^. The UK government recognises that current land use is not sustainable and that there is now an unprecedented opportunity to define a better land strategy that responds fully to the interconnected challenges of climate change, biodiversity loss and sustainable development^75^. We therefore need to make the land work harder for nature and move towards a resilient and sustainable future for Britain that benefits both people and nature.

With accelerating extinction rates and changing bioclimates, there is a pressing need to identify and implement effective actions for reversing biodiversity loss^9,11,12^, including at national scales. Some studies have looked at actions directly^9,76,77^. But these analyses can be sensitive to missing variables (e.g., confounding effects^76^), cross-sectoral impacts^78^, unrealistic outcomes (e.g., anti-environment attitudes coupled with widespread conservation action), or displacement of impacts (i.e., leakage). By contrast, here we investigate scenarios across a range of sectors and drivers within a European^36^ and global^20^ context. These scenarios, therefore, form coherent packages of actions across a range of attributes. Thus, we use explorative scenario modelling to provide a diverse range of options for policymakers.

The emergence of novel bioclimates over the coming decades presents enormous conservation and management challenges. As new combinations of species come into contact in new ways, some biotic interactions may break down while others may form, leading to potentially cascading effects^39,79^. In this new world, traditional nature recovery goals, such as place-based conservation^80^, could be ineffective^13,81^. Instead, shifts towards more open-ended, adaptive and dynamic approaches may be required^82,83^. Decisions may need to shift from resisting sometimes inevitable change to the facilitation of positive change for people and biodiversity^13,81^, which could include more widespread implementation of approaches such as assisted colonisation^84,85^. Indeed, the recent past is not always a reliable guide to future change, and conservation must therefore look to the future if it is to successfully anticipate and mitigate biodiversity loss^8^. In response, there have been calls for resilience^82,86^ or complexity^87^ focused approaches, which can foster adaptation to future climate impacts by restoring dynamic processes that promote natural variability within ecosystems and multifunctionality, and reduce the risk of dramatic ecosystem change^82,87^.

Our data-led analysis highlights the potential for the rearrangement of biodiversity, the disappearance of existing bioclimates, the emergence of novel bioclimates, and the accumulation of a sizeable extinction debt across Britain into the future. Our analyses illustrate that it is not too late to limit biodiversity change, but that this window is closing – actions taken during the next 20 years are crucial to foster more positive outcomes for British biodiversity. It might be that these time lags (i.e., extinction debt) provide a buffer for some species, during which time climate change might slow, species might adapt, or conservation efforts might succeed^40^. However, the rate of payment of extinction debts and the threat posed by novel and disappearing bioclimates suggest the need to plan for adaptation actions to address inevitable biodiversity losses^85^. Moreover, to achieve biodiversity recovery requires broad engagement, dialogue, and action across scales and sectors^88^. Indeed, policy and action now will determine whether Britain will meet its national biodiversity targets, as well as its international obligations through the Paris Agreement^89^ and the GBF^12^. Effective policy and action rely on a strong evidence base. We suggest that our national quantitative analysis, which provides high-resolution contextual information that is challenging to achieve within global analyses, makes a major contribution to evidence-based understanding across Great Britain.

## Methods

Here, we explored the magnitude and spatial distribution of biodiversity change under a range of climate and land-use scenarios across Britain up to the year 2080. We coupled structured biological data (1002 plant species, 56 butterfly species, and 219 bird species) with environmental data in a compositional dissimilarity modelling framework. We then projected multiple beta diversity metrics (compositional change, disappearing bioclimates, and novel bioclimates) in response to four future climate scenarios (RCP2.6, RCP4.5, RCP6.0, and RCP8.5). We also combined these compositional models with projections of habitat condition, capturing climate and land-use driven changes in habitat quality under six plausible futures (RCP2.6-SSP1, RCP4.5-SSP2, RCP4.5-SSP4, RCP6.0-SSP3, RCP8.5-SSP2 and RCP8.5-SSP5), to derive estimates of native British species heading for extinction. The baseline is set to 2015, reflecting the baseline land use map, but more generally applies to the period 1980–2020 to which the scenarios are anchored.

These projections can help to improve our understanding of the range of possible outcomes for biodiversity, alert decision-makers to potential undesirable future impacts, and enable exploration of the effectiveness of policy options and management strategies across Britain.

We used R version 4.4.0^90^ for all our analyses. See Zenodo for R code summarising the major analytical steps: 10.5281/zenodo.14834251. We used multiple R packages (and their dependencies) for data preparation, analysis, and visualization, including blme 1.0-5^91,92^, BRCmap 0.11.0.1^93^, corrplot 0.95^94^, cowplot 1.1.3^95^, dplyr 1.1.4^96^, gdm 1.6.0-4^97^, gghalves 0.1.4^98^, ggplot2 3.5.1^99^, gower 1.0.1^100^, gstat 2.1-1^101,102^, iNEXT 3.0.1^103,104^, janitor 2.2.0^105^, lme4 1.1-31^106,107^, ncdf4 1.22^108^, pbmcapply 1.5.1^109^, raster 3.6-30^110^, rphylopic 1.5.0^111,112^, scico 1.5.0^113^, sf 1.0-16^114^, sp 2.1-4^115^, terra 1.8-5^116^, tibble 3.2.1^117^, tidyr 1.3.1^118^.

### Structured biological monitoring data

Standardised community survey data are the most appropriate type of biological data to use in modelling compositional dissimilarity^26,119^. These surveys generate abundance and/or incidence (i.e., presence-absence) data and therefore avoid or reduce many of the biases associated with presence-only data^120^.

We collated structured record data for plants^30^, butterflies^31^, and breeding birds^32^. These groups represent different trophic levels/functional groups, from primary producers (most plants) to primary consumers (most butterflies) to secondary consumers (most birds).

For plants, we used data from the National Plant Monitoring Scheme (NPMS)^30^. NPMS started in 2015 and is a habitat-based plant monitoring scheme across the UK where observers record plant occurrence across 1 km grid cells using small plots^121^. NPMS has three nested levels of survey that volunteers can participate at depending on their confidence, knowledge and their time available^121^. Here, we chose to extract data from 2015 to 2021 at the inventory level (all vascular plants are recorded) to ensure the data reflect presence-absence samples (i.e., the inventory level is the only level where we know that unrecorded species were unobserved rather than not searched for).

For butterflies, we used data from the UKBMS, a UK survey where observers undertake standardised counts of butterflies. The main method used is the butterfly transect, a fixed route along which all butterflies are identified and counted weekly for up to 26 weeks a year, from April to September, to cover the peak flight periods of all UK butterflies^122^. We obtained annual estimates of the relative abundance of each species, adjusted for missing counts by integrating the species’ seasonal phenology^123,124^, for all transects surveyed between 2001 and 2010 (site index^31^). We included data for 57 regularly occurring British butterfly species (due to difficulties in field identification, we used aggregated records of Essex Skipper *Thymelicus lineola* and Small Skipper *T. sylvestris*). We degraded the abundance data to incidence (presence-absence) data. To allow spatial analyses, we assigned these UKBMS transects to 1 km grid cells based on their geolocation, merging the biological data where necessary (e.g., if more than one transect fell in the same cell).

For birds, we obtained count data from the UK Breeding Bird Survey (UK BBS)^32^, a nationwide survey in which experienced volunteers walk two 1 km transects across a 1 km grid cell^125^. UK BBS selects 1 km cells using a stratified random sampling design. Each cell is surveyed annually in two early morning visits during the breeding season (early visit: beginning of April to mid-May; late visit: mid-May to end-June). We only included species listed as category A in the British List (10th Edition)— “species that have been recorded in an apparently natural state at least once since 1 January 1950”^126^. We extracted maximum counts (across the early and late visit) per species from cells that had been surveyed between 2002 (UK BBS having been severely disrupted by the foot-and-mouth disease outbreak in 2001) and 2010. Counts were included from distance bands 1 to 3 (0–25 m, 25–100 m, >100 m); flyovers were excluded. Counts were then degraded to incidence (presence-absence) data.

The biological data varies in sampling effort (differing number of years surveyed per grid cell). Researchers have generally discounted the potential impact of unequal sampling effort or unequal sample coverage on compositional dissimilarity. Yet there is potential for bias (e.g., undersampling bias—not recording all taxa present at a site) that can lead to unreliable dissimilarity estimates, especially for incidence-based analyses^127^. To investigate and account for unequal sampling effort across our datasets, we quantified sample coverage – a measure of sample completeness^103^. Quantifying sample coverage provides a more direct assessment of the completeness of the biological data^103,128^, compared to alternative approaches, such as using equal-sized samples (e.g., downsampling more frequently recorded sites to match those sampled more infrequently^129^), or using species richness to adjust for variation in completeness^26,130^. Using our prepared biological data, we estimated sample coverage for each taxonomic group per grid cell, using the iNEXT function from the iNEXT package^103,104^. In this step, we removed any cells where sample coverage could not be reliably estimated (e.g., cells with less than two sampled years). Sample coverage was generally high for most grid cells (Supplementary Fig. 15). We weighted grid cells in our GDMs, with the formatsitepair function in the gdm package^97^, by our estimates of sample coverage to account for differences in sampling between grid cells.

The biological data were therefore prepared into incidence data for each of the taxonomic groups per grid cell per year. Overall, we prepared biological data for 1002 plant species (~72% of British native plant species) across 253 grid cells, for 56 butterfly species (~97% of British native butterfly species) across 926 grid cells, and for 245 breeding bird species (~96% of British native breeding bird species) across 4445 grid cells (Supplementary Fig. 16).

### Environmental data

We selected environmental variables a priori, based on their known or expected association with biodiversity and community composition. In particular, we built on the set of predictors used in previous models of compositional dissimilarity^23,131^. Our selected variables included two topographic variables, one soil variable, and seven climatic variables (Supplementary Fig. 17). We included topographic ruggedness index, topographic wetness index, soil pH, annual minimum temperature, annual maximum temperature, maximum monthly diurnal temperature range, annual precipitation, precipitation seasonality (coefficient of variation), potential evapotranspiration with interception correction (PETi) of driest month, and surface downwelling shortwave radiation (i.e., sunshine hours). We reprojected and then resampled data to a 1 km British grid where applicable.

Topographic ruggedness index (the mean of the absolute differences in elevation between a focal cell and its 8 surrounding cells) reflects changes in elevation, and was extracted at 1 km^132^. Topographic wetness index captures terrain-driven variation in soil moisture, which impacts plant growth, distribution and community assembly, and was extracted at ~450 m resolution^133,134^.

We obtained modelled estimates of soil pH at 1 km^135^. These estimates are based on soil pH data from 2446 locations across Britain and are representative of 0–15 cm soil depth. Estimates were available for 90% of grid cells; however, for the other 10%, we interpolated values using inverse distance weighting with the gstat package^101,102^.

We selected climate variables to represent the major dimensions of climate for biodiversity^23,131^. We extracted directly, or calculated (e.g., we calculated the maximum monthly diurnal temperature range from the difference between monthly maximum and monthly minimum temperature), the mean for multiple climate variables across the years 1980–2080 at 1 km resolution^27,136^. We used bias-corrected climate estimates (i.e., corrected to ensure consistency with the historical climate baseline) from ensemble member “01”.

### Future climatic data

We also prepared climatic data for four climate scenarios (the other environmental variables were treated as static). Specifically, we used yearly climatic projections^27,136^ under RCPs 2.6, 4.5, 6.0, and 8.5, taking the mean value for each of the seven climatic variables (see above) across three forecast periods: 2020–2040, 2040–2060, 2060–2080. We used UK-specific RCPs that are globally consistent^27^ (here applied to Britain rather than the UK because consistent biological data were not available for Northern Ireland). These climate projections are based on a nested Regional Climate Model (RCM) for the UK, specifically the UK Climate Projections 2018 (UKCP18; see ref. ^27^). Such RCMs perform atmospheric analyses for specific geographical domains at resolutions significantly smaller than those of the Earth System Models (ESMs) that are the primary approach to estimating climate change. One caveat of our analysis is that these fine-resolution projections are embedded in a single ESM, meaning that uncertainties in the evolution of future climate are not fully captured in our projections of biodiversity changes. We also acknowledge that while we employed the primary UKCP18 simulation available (ensemble member “01”), there are additional UKCP18 simulations that correspond to perturbed parameters, which are designed to capture some of the differences among the range of available ESMs. However, our fine-scale biodiversity calculations are extremely computationally intensive (~2.9 million cumulative computing hours), and as such, we have not repeated our analysis for the complete set of UKCP18 ensemble climate forcing data.

### Future land-use data

We used published land-use projections for Britain^28,137^. These land-use projections are based on a new agent-based model of the British land system, CRAFTY-GB^28^, and are available at 1 km resolution for the focal period with a 2015 baseline and decadal projections 2020–2080^137^. These projections model the same RCP-SSP plausible futures as we applied here (Supplementary Table 1), capturing climatic and socio-economic scenarios in the British land system. Specifically, they capture changes in societal preferences for ecosystem services (demand and valuation), individual and social behaviours among land managers, productive potential through a range of human and natural capitals and forms of land management, and changes in political and governance structures^28^. The projections are embedded within global simulations of the same sets of scenarios to ensure consistency across scales, including through the UK’s trade with other countries.

### Compositional dissimilarity

Underpinning our biodiversity projections, we built models of compositional dissimilarity (Fig. 1). Specifically, we used GDMs^130^. GDMs have been shown to offer advantages over SDMs when projecting the impact of climate change under non-analogue climate conditions^138^. In addition, GDMs consider communities of species in combination, and therefore implicitly include species interactions (compared to stacked SDMs, which do not^139–141^). As well as requiring fewer parameters to model community responses and therefore allowing rare species to be included^26^.

GDMs relate compositional dissimilarity (i.e., beta diversity) between pairs of sites to differences between sites in environmental and/or geographic distance^26,130^. GDMs accommodate two types of nonlinearities that are common in ecological datasets: (1) the variation in the rate of compositional change (non-stationarity) along environmental gradients, and (2) the curvilinear relationship between biological distance and environmental/geographical distance. To address non-stationarity, GDMs first use maximum-likelihood estimation and flexible I-splines (here, the default of three knots, positioned at the minimum, median and maximum) to transform each of the environmental variables and identify the best supported relationship linking environmental separation and compositional dissimilarity between sites. Second, this scaled combination of distances between sites is transformed via a negative exponential link function to accommodate the curvilinear relationship between compositional dissimilarity (here Jaccard similarity) and environmental separation. For more details, see refs. ^26,130^.

We built GDMs for each taxonomic group at 1 km resolution, based on the prepared species composition data (see above) and ten environmental variables (see above) using the gdm function from the gdm package^97^. Prior to modelling, we checked for collinearity among covariates. All pairwise Pearson correlations were <0.8, except for annual maximum temperature and annual minimum temperature (0.85), which are strongly correlated as expected. GDMs are recognised as robust to multicollinearity^142^, so we retained both temperature variables to represent distinct aspects of climate variation, consistent with previous analyses^23,131^. This approach also reflects our primary aim of representing ecological relationships rather than interpreting individual variable effects. These GDMs therefore characterised the species-environment relationships for the baseline period (Supplementary Figs. 18–20). The baseline GDMs showed good fit (plants = 32% explained deviance, butterflies = 31%; birds = 56%; Supplementary Fig. 21), with GDMs generally having low explained deviance^26^, e.g., 20–50%.

### Biodiversity projections

Using our baseline GDMs, we made biodiversity projections for multiple metrics (compositional change, disappearing bioclimates, novel bioclimates, species heading for extinction). To enable these biodiversity projections, we first transformed the prepared future climate data with the gdm.transform function^97^. This function transforms the future climate variables based on the modelled ecological relationships characterised by the baseline GDMs. Specifically, the function provides a non-linear transformation of each climate variable to include the estimated coefficients^143^. This approach relies on space-for-time substitution. In other words, the spatial variation in species composition observed under current climate can be used to predict variation in composition through time under changing climates. There is evidence that such an assumption can be reasonable when modelling compositional similarity^144^.

### Compositional change

We then quantified compositional change—the magnitude of compositional dissimilarity between time points in response to climate change under the four RCPs. To calculate compositional change, we used the transformed climate variables. We then predicted change through time (using the predict.gdm function with argument time = TRUE^97^), removing the intercept from the predictions^26^. If we did not remove the intercept from the predictions, the results would reflect an assumed difference in composition for a grid cell over time, even if environmental conditions remained constant. This could be realised through stochastic processes, but here we more conservatively assume no change through time if the environment does not change. In practice, compositional change will take place through processes not explicitly modelled here: dispersal in and out of the area, and local adaptation^23^.

### Disappearing and novel bioclimates

We also quantified the spatial distribution of disappearing and novel bioclimates^26^. Where bioclimates reflect the integration of biological and climatic components through the GDM process. That is, rather than describing a location solely by its climate^37,145^, we describe a location by its climatic ability to support predicted compositions. Although strictly going beyond climate and including multiple environmental dimensions (topography, soil), only the climatic variables change through time under the four RCPs, with the other environmental variables remaining static; thus, we refer to bioclimates for clarity. We use the more conservative term bioclimates^146,147^ rather than ecosystems^148^, as the extent and structure of the true ecosystems is unknown, and rather than environments^26^, as this term has a loose definition and is often used to refer only to the abiotic components of systems.

In the context of our bioclimates, analogues are defined as current bioclimates that are <10% dissimilar (>90% similar) to any future bioclimate. We selected a dissimilarity threshold of 10% as it reflected a balance where not every pair of bioclimates was identified as different, but was still sensitive enough to identify analogue bioclimates^38^. By contrast to analogues, disappearing bioclimates are current bioclimates that are ≥10% dissimilar to any future bioclimate (forward looking). Essentially, disappearing bioclimates are bioclimates that exist today but do not closely match future bioclimates. Novel bioclimates are future bioclimates that are ≥10% dissimilar to any current bioclimate (backwards-looking). Hence novel bioclimates are not found across Britain today but emerge under the future climate scenario. Thus, novel bioclimates capture the emergence of species-climate combinations without present-day analogues.

To calculate disappearing bioclimates, we predicted the dissimilarity between a current focal cell (${i}_{{{{\rm{current}}}}}$) and all $n$ future cells (${j}_{{{{\rm{future}}}}}\in \{1:n\}$) with the predict.gdm function and time = FALSE^97^, removing the intercept from the predictions^26^. For novel bioclimates, we predicted the dissimilarity between a focal future cell (${i}_{{{{\rm{future}}}}}$) and all $n$ current cells (${j}_{{{{\rm{current}}}}}\in \{1:n\}$). The quantification of disappearing and novel bioclimates depends on both the magnitude of change (Supplementary Figs. 2–4), as well as the frequency of occurrence of different bioclimates (e.g., currently scarce bioclimates are more likely to disappear, all else being equal). We used all cells for these calculations (one combination of a taxonomic group, an RCP, and a time horizon took ~40 computing hours to run).

### Species heading for extinction

Finally, we estimated the percent of species persisting, as a function of climate change and land-use change, and the species-area relationship^23,149^ (Fig. 1). Species persisting is the percentage of species originally occurring in each grid cell expected to persist anywhere in Britain over the long term given habitat loss/modification^26^. Where the complement to species persisting is species heading for extinction (also referred to as committed to extinction^23,33^). In brief, this approach scales the availability of remaining habitat across all grid cells with a similar bioclimate to a focal grid cell, relative to the area of this bioclimate that would be present in an intact landscape^23^. This approach has been applied previously^23,149,150^ and is recognised by the Biodiversity Indicators Partnership (BIP) as the Biodiversity Habitat Index (https://www.bipindicators.net/indicators/biodiversity-habitat-index).

### Plausible futures

We calculated species heading for extinction for six plausible futures (i.e., scenarios constrained by realistic and credible trends based on current knowledge) defined by plausible combinations of the four RCPs (i.e., changes in climate; see above) and five SSPs (i.e., changes in socioeconomic conditions) (Supplementary Table 1). We used UK-specific SSPs that are locally comprehensive, yet consistent with the global and European SSPs^29,151,152^. These UK SSPs integrate bottom-up stakeholder knowledge and locally-relevant drivers with top-down information from the global and European SSPs, via a professionally facilitated participatory process^151^. Thus, the UK SSPs are embedded within the global and European SSPs but include extensions beyond them to incorporate UK-specific drivers^151^. Here we describe the SSPs in brief (Supplementary Table 2)^19,28,29,153^, but for more detail see ref. ^29^, or for videos and fact sheets describing the SSPs, see ref. ^154^.

The RCP-SSP framework has been adopted across disciplines, as it provides coherent storylines of plausible future conditions^20,28^. Hence, we crossed UK RCPs^27^ with UK SSPs^29,151,152^ to create plausible futures—plausible and often simplified descriptions of how the future may develop based on a coherent and internally consistent set of assumptions about key driving forces and relationships. Specifically, we explored RCP2.6-SSP1, RCP4.5-SSP2, RCP4.5-SSP4, RCP6.0-SSP3, RCP8.5-SSP2, and RCP8.5-SSP5 (Supplementary Table 1). Together, these RCP-SSP plausible futures describe radically different worlds^28^.

### Habitat condition

To estimate the number of species heading for extinction for the six plausible futures, we required maps of habitat condition (the proportional species richness expected to be retained per grid cell)^23,26^. Building on a global scale approach^23^, we generated estimates of habitat condition for Britain by combining projections of land use^28^ with modelled estimates of the Biodiversity Intactness Index (BII) for each land-use class (Fig. 1). Overall, BII combines two components: overall organism abundance relative to a reference condition and compositional similarity to a reference assemblage^155–157^ (details below).

We estimated BII for different land-use classes by analysing data from the PREDICTS database^158^—a global collation of studies of multiple sites differing in land use. There were insufficient data available for Britain alone within the PREDICTS database, so we selected sites in all temperate biomes (Temperate Conifer Forests, Temperate Broadleaf & Mixed Forests, Temperate Grasslands, Savannas & Shrublands, Mediterranean Forests, Woodlands & Scrub), for a total of 19,828 sites.

To define a reference condition (also known as a baseline), we selected sites from the PREDICTS database that represent minimally and lightly used primary vegetation, and minimally and lightly used mature and intermediate secondary vegetation (7067 sites; Supplementary Table 3). These reference sites reflect a natural/semi-natural state against which other land uses are compared. This inclusive definition (beyond strictly pristine primary vegetation) likely elevates our BII coefficients, leading to more conservative estimates of species heading for extinction. Nevertheless, these sites provide a robust representation of natural and semi-natural conditions.

To estimate BII, we fit two mixed-effects models^156,157^. The first model captured the total abundance of organisms, calculated as the sum of abundance across all species recorded at each site. Where sampling effort varied among sites within a study, and abundance was reported in an effort-sensitive metric (e.g., counts), we standardised by dividing abundance by sampling effort^157,159^. Within each study, we then rescaled abundance so that the maximum value equalled one, reducing inter-study variance from methodological and taxonomic differences^157^. We square-root transformed abundance and modelled it using Gaussian errors because non-integer abundance measures precluded modelling of untransformed data with Poisson errors, and square-root transformation resulted in better residual distribution than log transformation^156^.

We modelled rescaled abundance as a function of the following fixed effects: land-use class (harmonised between the PREDICTS database^158^ and the CRAFTY land-use projections^28,137^; Supplementary Table 3) and net primary productivity (NPP; square-root transformed). We included NPP as a control variable to account for agricultural sites typically occurring in high-NPP areas. We obtained NPP values for each site from MODIS^160^ at 500 m resolution for the year in which sampling began. We also included random intercepts for study identity and spatial block within study to account for differences in sampling methodology, large-scale environmental variation, and spatial structure within studies^156^.

The second model assessed compositional similarity between sites, capturing changes in community composition relative to the reference condition. For studies with at least one reference site, we calculated compositional similarity as 1–d_BC-bal_ between each reference site and each other site in turn^157^, where d_BC-bal_ is the balanced variation component of the (corrected) abundance-based Bray-Curtis dissimilarity metric^161^. This measure essentially reflects the overlap in species abundance distributions between the two compared sites^162^. If either site in the comparison had no individuals, we set compositional similarity as 0. We logit transformed compositional similarity prior to analysis, applying an adjustment of 0.05 to accommodate zeros and ones.

We modelled compositional similarity as a function of the following fixed effects: land-use transition (reference vs other land-use classes), geographic distance (log-transformed), environmental distance (Gower dissimilarity based on climate and elevation, cube-root transformed), and NPP (square-root transformed). We included third-degree orthogonal polynomials for geographic and environmental distance to allow for non-linear decay in compositional similarity with increasing distance. We calculated geographic distance between sites and divided by the average size of a sampling plot in the data set. We calculated environmental distance using WorldClim^163^ variables (minimum temperature of the coldest month, precipitation of the wettest and driest months, and elevation) using the gower package^100^. Like the abundance model, we included NPP as a control variable to account for agricultural sites typically occurring in high-NPP areas. We included random intercepts for study identity and for the identity of the second site in each pairwise comparison to remove pseudoreplication that would otherwise arise from comparing each reference site to every other site within the study.

We fit the abundance model using the lmer function from the lme4 package^106,107^. We fit the compositional similarity model with the blmer function from the blme package^91,92^ using the bobyqa optimizer^164^, to improve convergence and stability in the presence of complex random-effects structures. For both models, we used Restricted Maximum Likelihood, variables were scaled and centred before modelling, and we assessed whether random slopes were required by assessing Akaike’s Information Criterion for models with each variable fit as a random slope in turn^165^. We calculated BII for each harmonised land-use class (Supplementary Table 4) by multiplying the respective abundance and compositional similarity coefficients together. These values represent the proportional species richness retained relative to the reference sites.

The urban BII coefficient was adjusted to account for impermeable areas of urban land use having a BII of zero, based on the mean urban imperviousness in the UK being 30%^166^. The agroforestry BII was constructed by averaging the BIIs of sustainable agriculture, extensive pastoral and productive forestry. In addition, under SSP5, two land uses—intensive pastoral and intensive agriculture—shift to “very high levels of intensification”^28^, levels not seen in the current day and beyond all other SSPs. These intensity values reflect a combination of the use of agricultural inputs (fertilisers, pesticides and machinery), technology, and modelled production levels^28^. We therefore created special land-use classes of hyper-intensive pastoral and hyper-intensive agriculture for SSP5. Through discussion, and based on the SSPs^152^ and land-use dynamics^28^, we decided to set the maximum BII of hyper-intensive pastoral as the BII of intensive pastoral plus 80% of the distance between extensive pastoral and intensive pastoral. We then made these BIIs dynamic, so that they changed in line with average intensity over the future period. Specifically, we linearly interpolated between the BII of intensive pastoral in 2037 (0.63; the point at which the intensity of pastoral becomes unprecedented in SSP5) to the maximum intensity in 2070 (0.50; after which intensity plateaus). We applied the same process to hyper-intensive agriculture. Overall hyper-intensive pastoral becomes 21% more intense than intense pastoral over the future period, and hyper-intensive agriculture becomes 11% more intense.

We applied our calculated BII estimates for each harmonised land-use class (Supplementary Table 4) to the CRAFTY land use projections^28,137^ for each grid cell, generating spatially explicit maps of habitat condition for Britain for each year. We then averaged these projected BII estimates across our focal time slices (2020–2040; 2040–2060; 2060–2080).

Overall, our approach follows the assumption that differences in condition caused by land-use change over time, at a single location, are comparable to the differences observed between land uses across different locations at a single point in time^23^, as assessed by models built using the PREDICTS database^158,167^.

### Calculating species heading for extinction

We estimated the proportion of species (${p}_{i}$) persisting in grid cell $i$ as (Eq. (1)):

1 $${p}_{i}={\left[\frac{{\sum }_{j=1}^{n}{{{\rm{Sim}}}}\left({i}_{{{{\rm{current}}}}},{j}_{{{{\rm{future}}}}}\right)\times {{{\rm{hab}}}}[{j}_{{{{\rm{future}}}}}]}{{\sum }_{j=1}^{n}{{{\rm{Sim}}}}\left({i}_{{{{\rm{current}}}}},{j}_{{{{\rm{current}}}}}\right)\times {{{\rm{hab}}}}[{j}_{{{{\rm{current}}}}}]}\right]}^{z}$$

where ${{{\rm{Sim}}}}\left({i}_{{{{\rm{current}}}}},{j}_{{{{\rm{future}}}}}\right)$ is the bioclimatic similarity between grid cell $i$ under the current climate and grid cell $j$ under the future climate and ${{{\rm{hab}}}}[{j}_{{{{\rm{future}}}}}]$ ($\le 1$) is the future habitat condition of grid cell $j$; ${{{\rm{Sim}}}}\left({i}_{{{{\rm{current}}}}},{j}_{{{{\rm{current}}}}}\right)$ and ${{{\rm{hab}}}}[{j}_{{{{\rm{current}}}}}]$ are the corresponding quantities under the current climate. The numerator represents the remaining extent of habitat in the future with bioclimates similar to that of the current cell $i$ (i.e., the amount of suitable habitat supporting the biological community originally present in cell $i$)^150^. The denominator represents the potential extent of similar bioclimates to a given cell $i$ under the current climate. Following standard practice, we assume that the current landscape is intact (as a benchmark to compare the future to) and therefore set ${{{\rm{hab}}}}\left[{j}_{{{{\rm{current}}}}}\right]=1$ for all grid cells^23^. The power exponent $z$ is the coefficient of the species-area relationship, set to the widely used value of 0.25^149^.

To make the analysis computationally tractable, we performed calculations of species heading for extinction with a random subsample of 20% of future grid cells (45,950 reference cells; randomly selected using slice_sample from the dplyr package^96^). This subsample ensures the representativeness of dissimilarity estimates by comparing each cell to a broad set of other cells, generating sufficiently precise values without the computational cost of evaluating all 26 billion (2.6 × 10^10^) possible pairwise comparisons. Each combination of taxonomic group, plausible future, and forecast period took ~50,000 cumulative processor hours when run in a high-performance computing environment (JASMIN^168^) using the 20% reference sample. We explored three taxonomic groups, six plausible futures, and three forecast periods, plus the baseline, totalling ~2.9 million cumulative computing hours. This figure represents total computational time; in practice, substantial parallel processing across multiple nodes reduced the actual runtime to ~1000 hours (approximately six weeks), making the analysis intensive but tractable.

The proportion of native species originally present across Britain expected to persist over the long term was then calculated as a weighted geometric mean across all grid cells^23^, the complement of which is species heading for extinction. We calculated a geometric mean, rather than an arithmetic mean, in keeping with recommendations regarding the appropriateness of this approach when aggregating relative, or proportional, measures of change across multiple elements of biodiversity^23,169^. Thus, beta diversity (models of compositional dissimilarity) and alpha diversity (species-area relationship) were combined to estimate changes in gamma diversity (species heading for extinction).

As for any species-area relationship based method^170,171^, this approach does not estimate the precise timing of extinction. Rather, the approach estimates the proportion of species which are expected to become extinct over the long term, as a consequence of the climate and land-use conditions predicted at a specified time point^23^. We therefore invoke the concept of species heading for extinction as those species that were originally present in an area and that are estimated to disappear from their entire range, given projected climate change and land-use change^150^. Some of these extinctions might be realised by the specified time point, whereas others are expected to be realised over longer time periods into the future, as an extinction debt^34,35,172^, unless habitat condition improves^150^. Further work is needed to better understand the rate at which these extinction debts are paid off^173,174^.

### Reporting summary

Further information on research design is available in the Nature Portfolio Reporting Summary linked to this article.

## Supplementary information

Supplementary Information

Reporting Summary

## Data Availability

All prepared and processed data are available at 10.5281/zenodo.14834251. Raw data are available from the original sources. Structured record data for plants: 10.5285/e742c94f-82a4-43e7-af14-36b131afe81b. Structured record data for butterflies: 10.5285/b77561d8-20ee-45ad-9221-7a8885d5ac8e. Structured record data for birds can be requested: https://www.bto.org/our-science/data/data-request-system. Topographic ruggedness index data: 10.1594/PANGAEA.867115. Topographic wetness index data: 10.5285/6b0c4358-2bf3-4924-aa8f-793d468b92be. Modelled estimates of soil pH: 10.5285/4b0e364d-61e6-48fb-8973-5eb18fb454cd. Climatic data: 10.5285/8194b416cbee482b89e0dfbe17c5786c. Land use projections: 10.5285/f9ab3051-4f85-415f-b691-371ff8e951f2. Biodiversity samples from the PREDICTS database: https://data.nhm.ac.uk/dataset/the-2016-release-of-the-predicts-database and https://data.nhm.ac.uk/dataset/release-of-data-added-to-the-predicts-database-november-2022. WorldClim data: https://worldclim.org/. Net primary productivity data: 10.5067/MODIS/MOD17A3HGF.061.

## Code availability

R code to reproduce the analyses is available at 10.5281/zenodo.14834251.
