# Supplementary material for: Future scenarios for British biodiversity under climate and land-use change
Source: Nat Commun. 2026 Mar 31;17:2704. doi: 10.1038/s41467-026-70064-4 (PMC13039725; doi:10.1038/s41467-026-70064-4)
Supplement: Supplementary file 1 — Supplementary Information [file 41467_2026_70064_MOESM1_ESM.pdf]

Future scenarios for British biodiversity under climate and land-use change

Supplementary Information

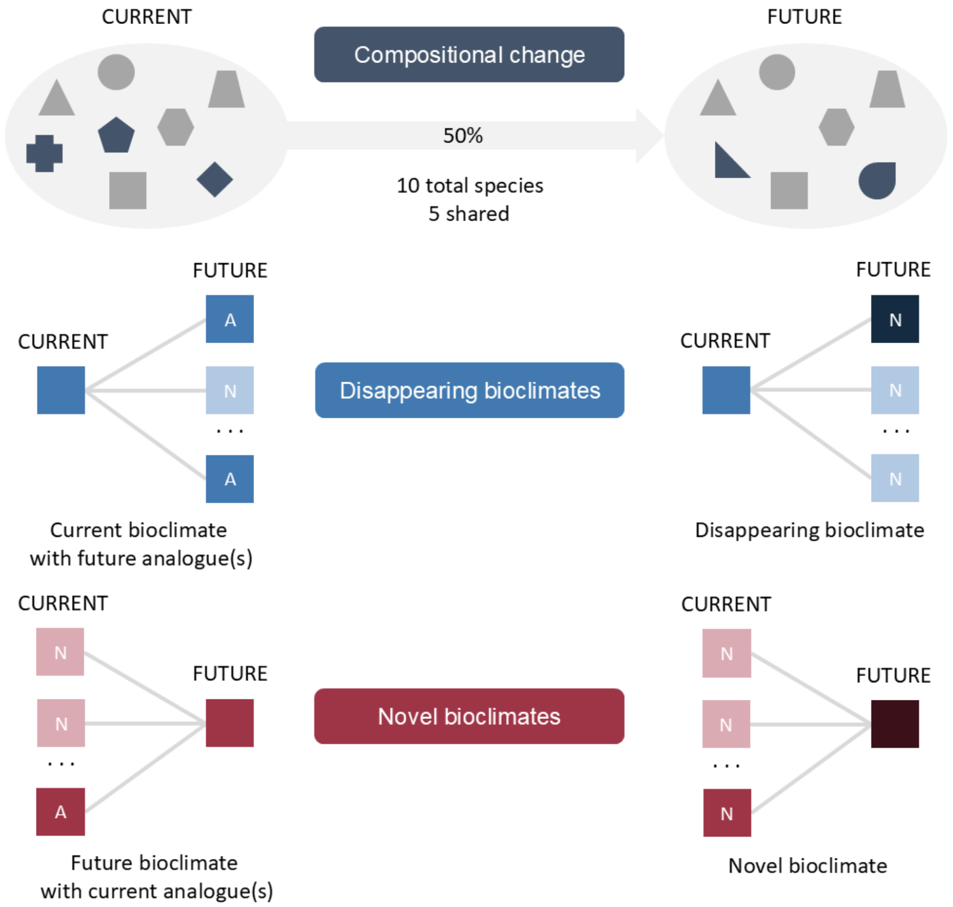

Supplementary Fig. 1: The three beta diversity metrics calculated. Compositional change is the magnitude of compositional dissimilarity between the present and future; different shapes represent different species; grey shapes are shared between the present and future. Disappearing bioclimates are current species-climate combinations with no future analogue, and novel bioclimates are future species-climate combinations with no current analogue. Squares represent grid cells; more similar colours indicate more similar bioclimates. A = analogue, N = non-analogue. Compositional change captures the reorganisation and reshuffling of species identities through time, whereas disappearing bioclimates capture the loss of currently observed species-climate combinations and novel bioclimates capture the emergence of species-climate combinations without present-day analogue.

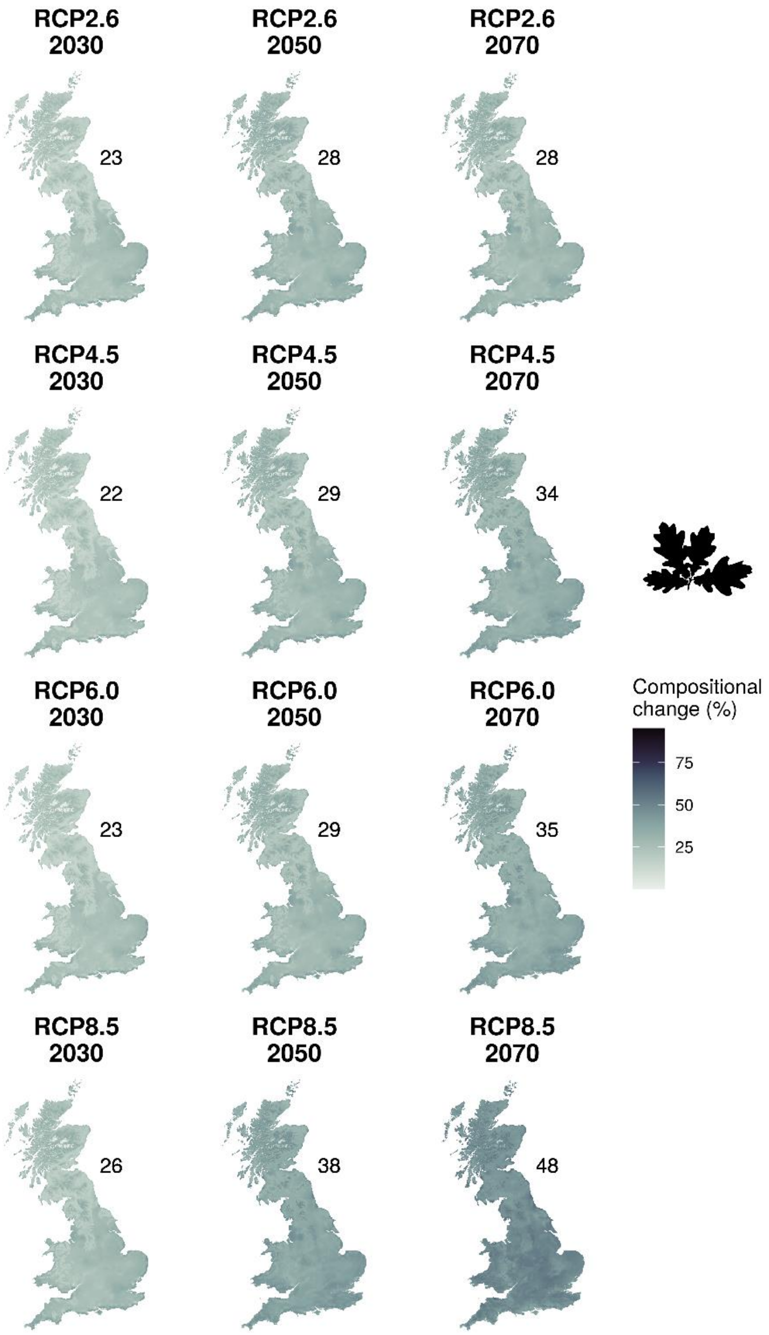

Supplementary Fig. 2: Compositional change for plants across Great Britain. Median compositional change (the percentage magnitude of compositional dissimilarity between the present and future) is summarised for four future climate scenarios (Representative Concentration Pathways; RCP) for the years 2020-2040 (centred on 2030), 2040-2060 (centred on 2050), and 2060-2080 (centred on 2070) for 1002 plant species. The scale is comparable across compositional change plots (Supplementary Figs. 2-4). The annotated numbers represent the median value across grid cells.

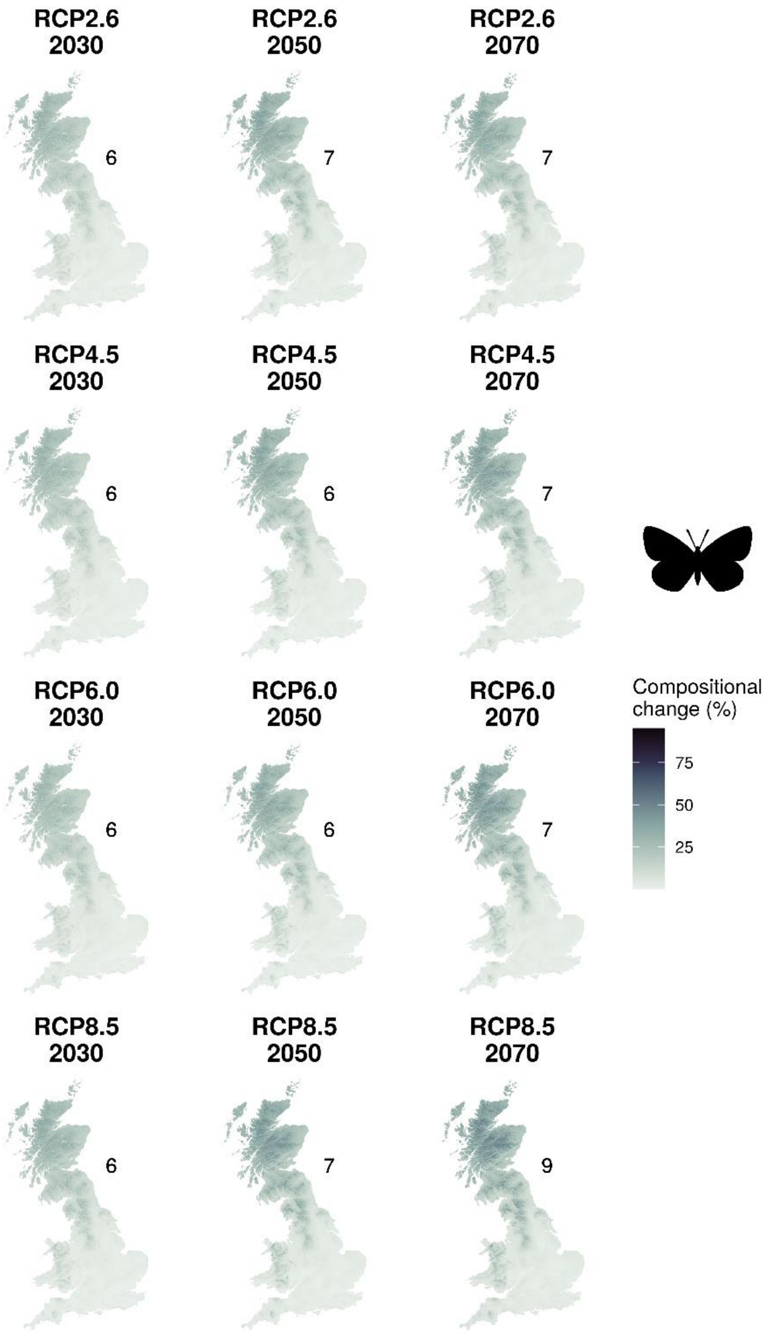

Supplementary Fig. 3: Compositional change for butterflies across Great Britain. Median compositional change (the percentage magnitude of compositional dissimilarity between the present and future) is summarised for four future climate scenarios (Representative Concentration Pathways; RCP) for the years 2020-2040 (centred on 2030), 2040-2060 (centred on 2050), and 2060-2080 (centred on 2070) for 56 butterfly species. The scale is comparable across compositional change plots (Supplementary Figs. 2-4). The annotated numbers represent the median value across grid cells.

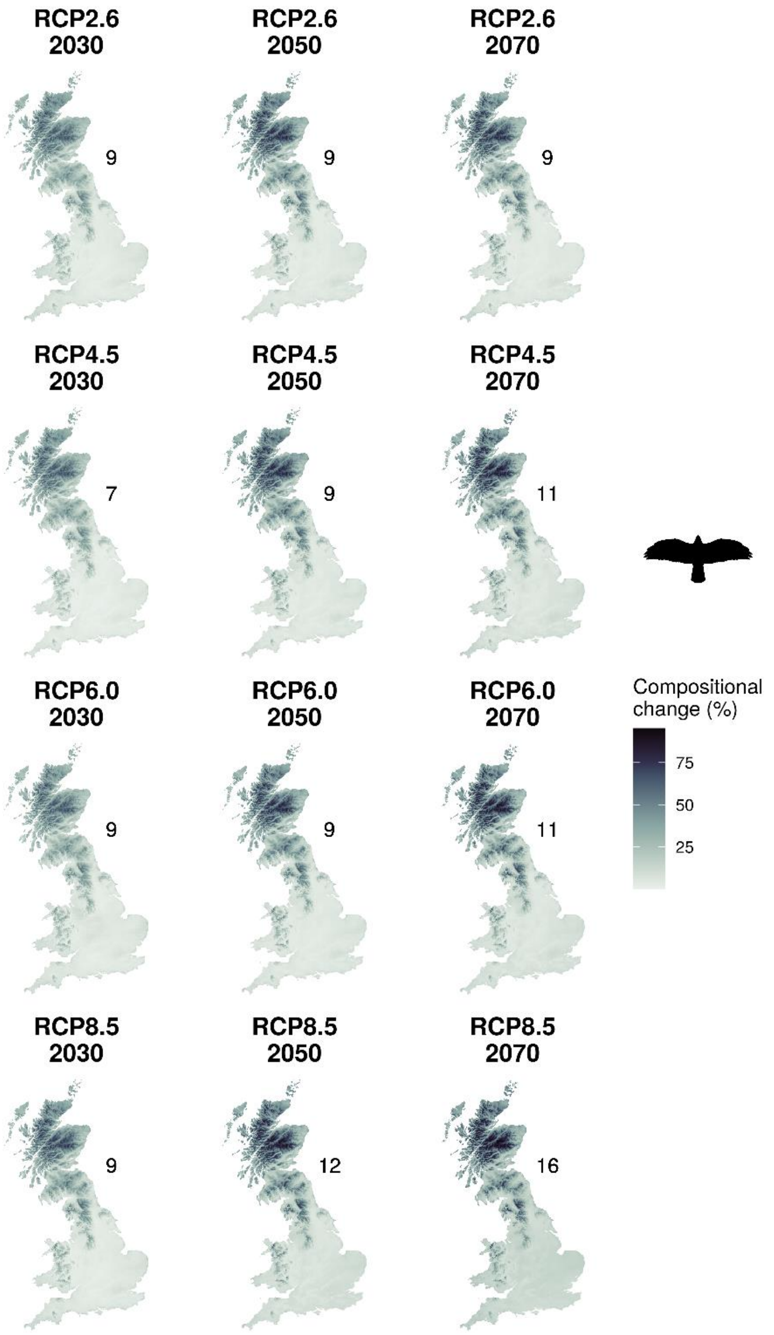

Supplementary Fig. 4: Compositional change for birds across Great Britain. Median compositional change (the percentage magnitude of compositional dissimilarity between the present and future) is summarised for four future climate scenarios (Representative Concentration Pathways; RCP) for the years 2020-2040 (centred on 2030), 2040-2060 (centred on 2050), and 2060-2080 (centred on 2070) for 219 bird species. The scale is comparable across compositional change plots (Supplementary Figs. 2-4). The annotated numbers represent the median value across grid cells.

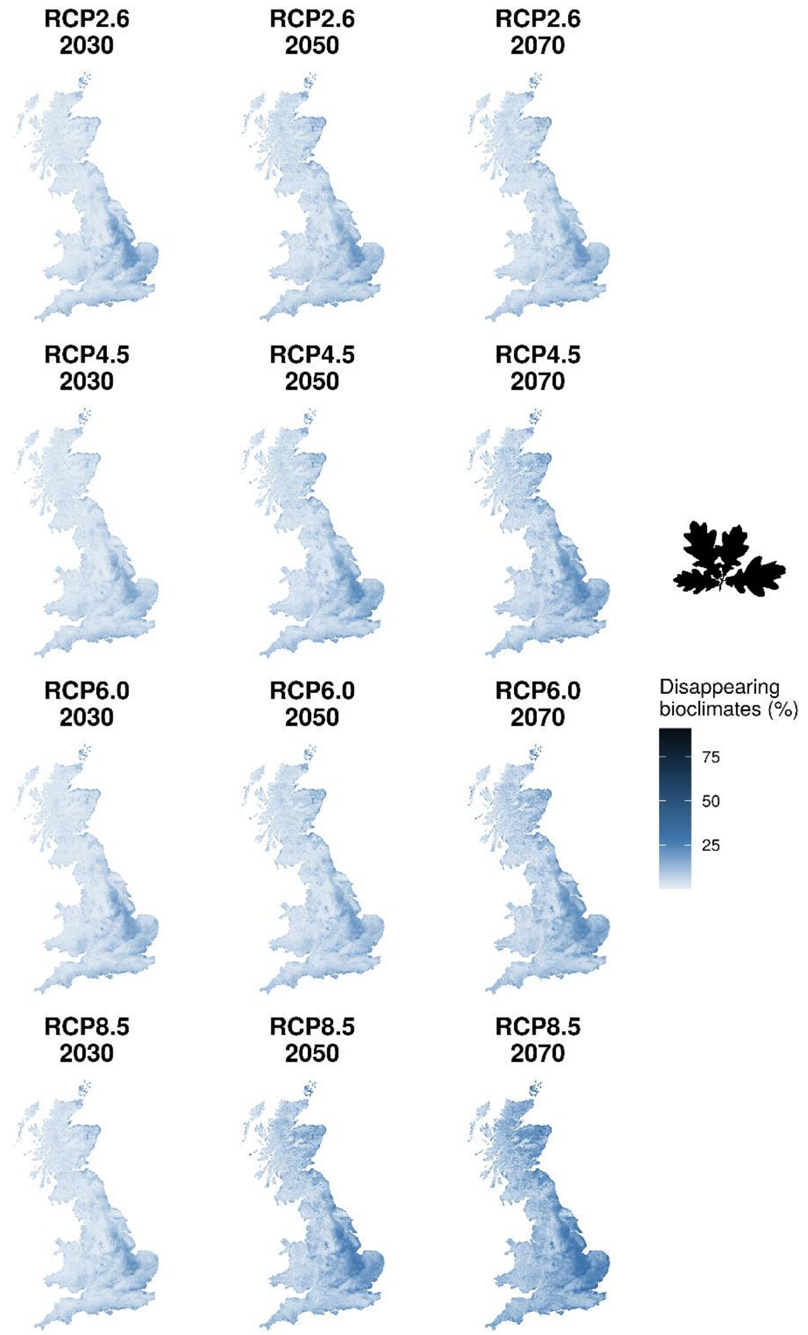

Supplementary Fig. 5: Disappearing bioclimates for plants across Great Britain. The dissimilarity of disappearing bioclimates (minimum dissimilarity between a grid cells' current bioclimate and all future bioclimates - i.e., how different is the most similar future cell) is summarised for four future climate scenarios (Representative Concentration Pathways; RCP) for the years 2020-2040 (centred on 2030), 2040-2060 (centred on 2050), and 2060-2080 (centred on 2070). The scale is comparable across disappearing plots (Supplementary Figs. 5-7).

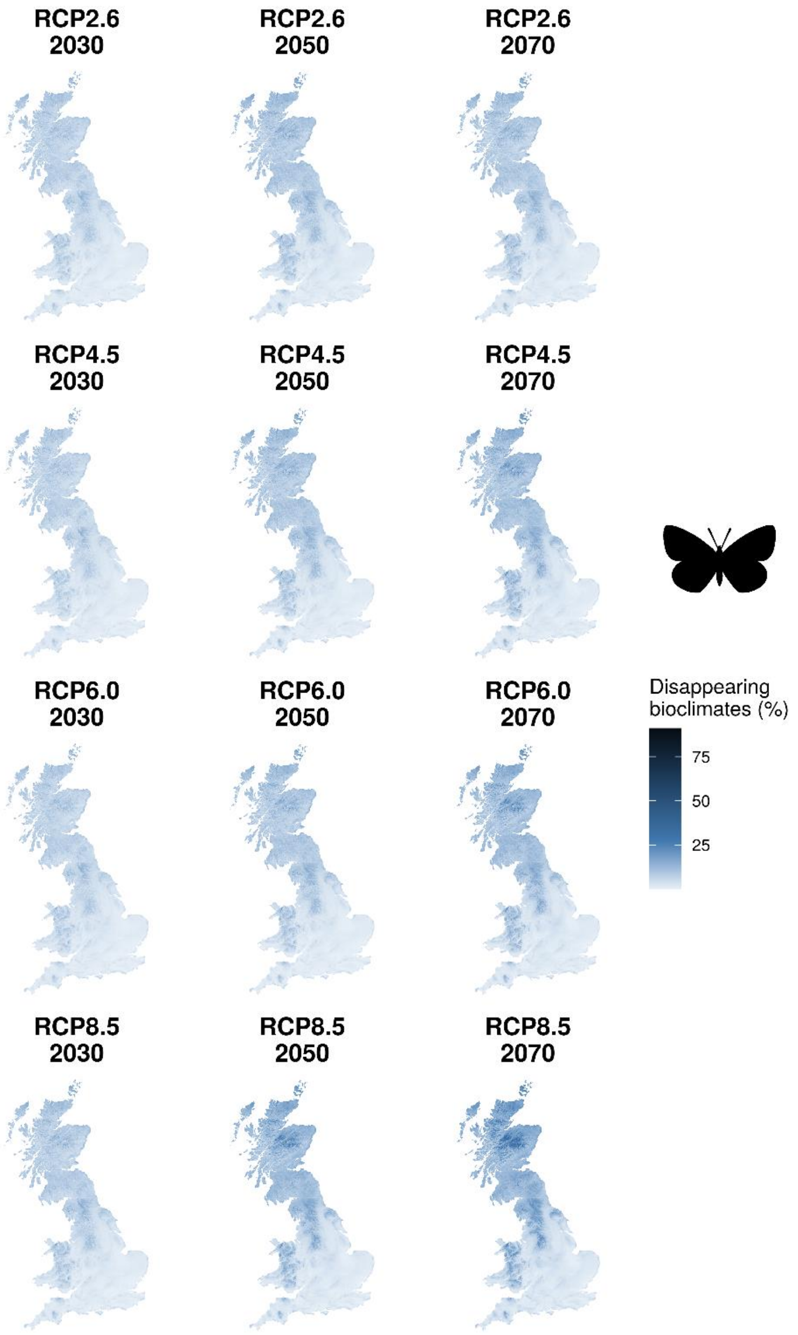

Supplementary Fig. 6: Disappearing bioclimates for butterflies across Great Britain. The dissimilarity of disappearing bioclimates (minimum dissimilarity between a grid cells' current bioclimate and all future bioclimates - i.e., how different is the most similar future cell) is summarised for four future climate scenarios (Representative Concentration Pathways; RCP) for the years 2020-2040 (centred on 2030), 2040-2060 (centred on 2050), and 2060-2080 (centred on 2070). The scale is comparable across disappearing plots Supplementary Figs. 5-7).

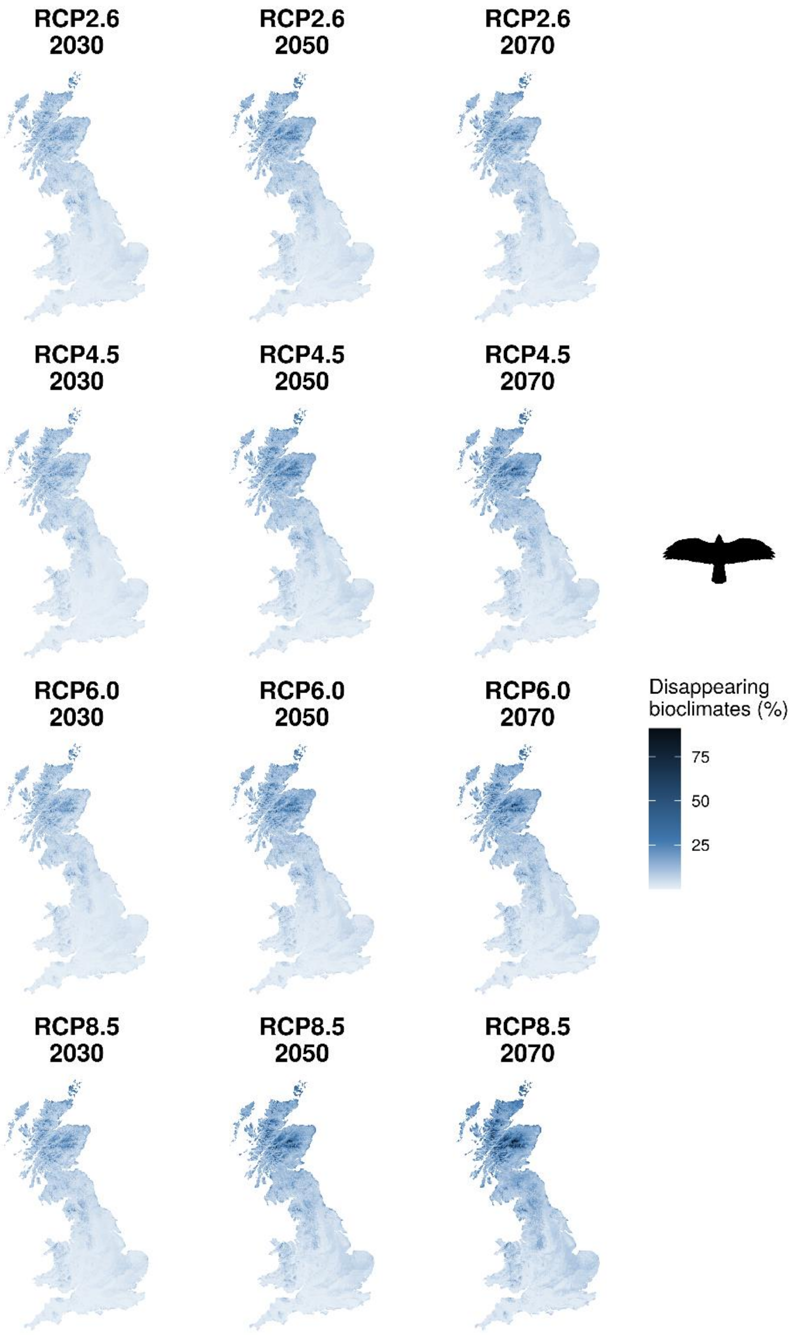

Supplementary Fig. 7: Disappearing bioclimates for birds across Great Britain. The dissimilarity of disappearing bioclimates (minimum dissimilarity between a grid cells' current bioclimate and all future bioclimates - i.e., how different is the most similar future cell) is summarised for four future climate scenarios (Representative Concentration Pathways; RCP) for the years 2020-2040 (centred on 2030), 2040-2060 (centred on 2050), and 2060-2080 (centred on 2070). The scale is comparable across disappearing plots (Supplementary Figs. 5-7).

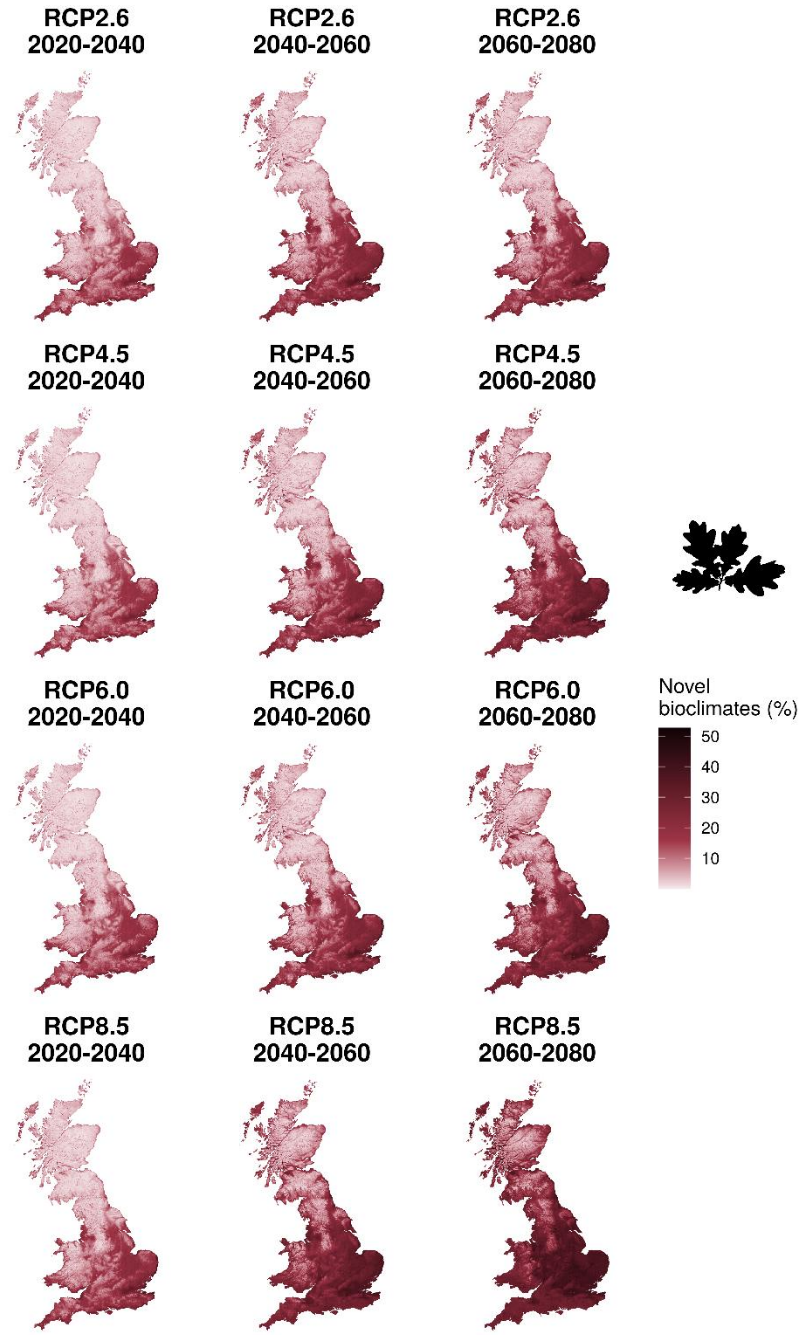

Supplementary Fig. 8: Novel bioclimates for plants across Great Britain. The dissimilarity of novel bioclimates (minimum dissimilarity between all current bioclimates and a grid cells' future bioclimate - i.e., how different is the most similar current cell) is summarised for four future climate scenarios (Representative Concentration Pathways; RCP) for the years 2020-2040 (centred on 2030), 2040-2060 (centred on 2050), and 2060-2080 (centred on 2070). The scale is comparable across novel plots (Supplementary Figs. 8-10).

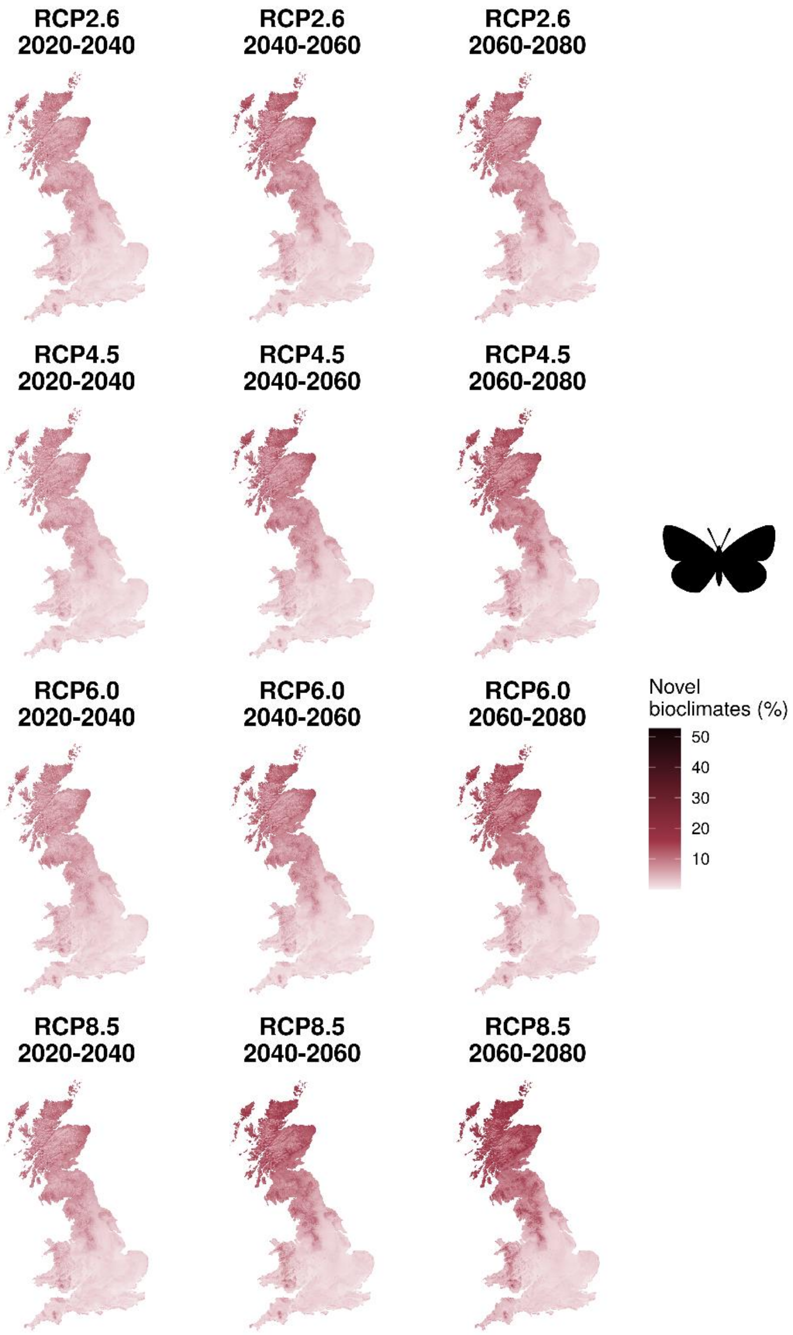

Supplementary Fig. 9: Novel bioclimates for butterflies across Great Britain. The dissimilarity of novel bioclimates (minimum dissimilarity between all current bioclimates and a grid cells' future bioclimate - i.e., how different is the most similar current cell) is summarised for four future climate scenarios (Representative Concentration Pathways; RCP) for the years 2020-2040 (centred on 2030), 2040-2060 (centred on 2050), and 2060-2080 (centred on 2070). The scale is comparable across novel plots (Supplementary Figs. 8-10).

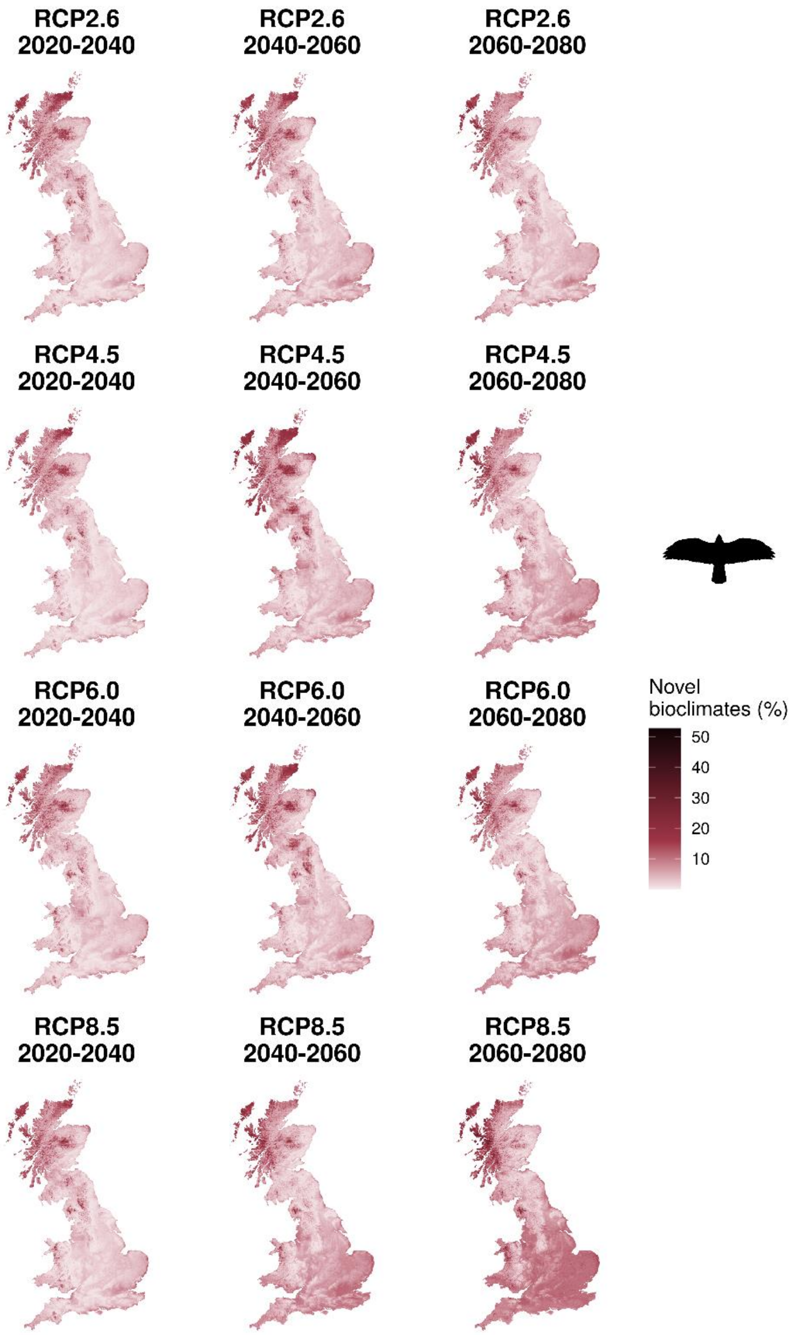

Supplementary Fig. 10: Novel bioclimates for birds across Great Britain. The dissimilarity of novel bioclimates (minimum dissimilarity between all current bioclimates and a grid cells' future bioclimate - i.e., how different is the most similar current cell) is summarised for four future climate scenarios (Representative Concentration Pathways; RCP) for the years 2020-2040 (centred on 2030), 2040-2060 (centred on 2050), and 2060-2080 (centred on 2070). The scale is comparable across novel plots (Supplementary Figs. 8-10).

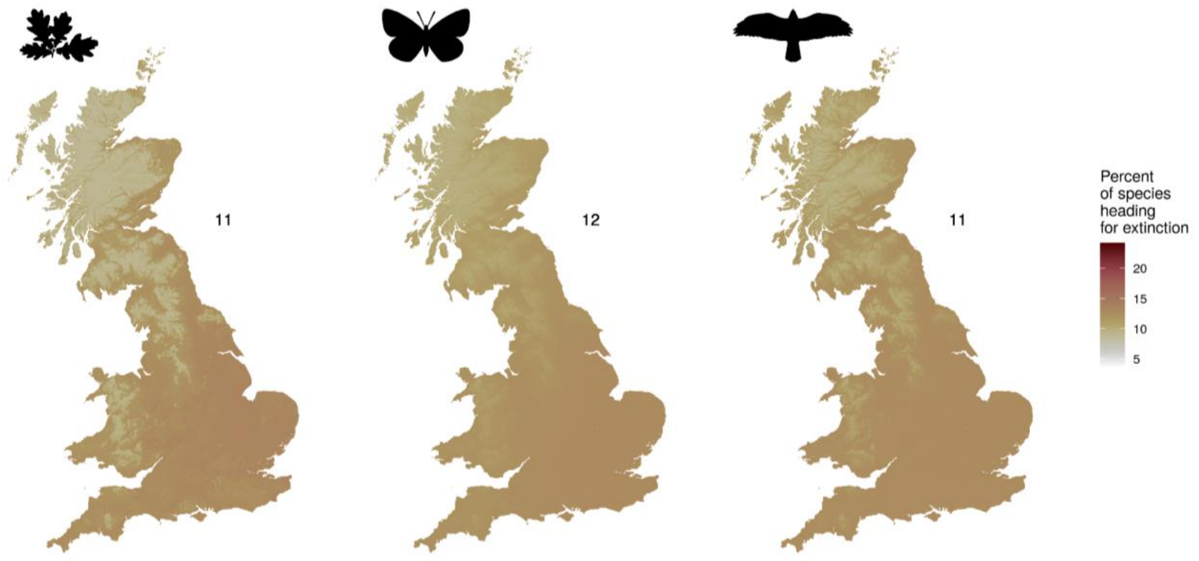

Supplementary Fig. 11: Species heading for extinction for the baseline period. The baseline period reflects the baseline land use map from the year 2015, but more generally applies to 1980-2020. The scale is comparable across extinction plots (Supplementary Figs. 11-14). The annotated numbers represent the weighted geometric mean across grid cells. The baseline is only in response to land-use change not climate change. All taxonomic groups have the same Biodiversity Intactness Index coefficients for land use (Supplementary Table 4) and the same spatial distribution of land use (i.e., land use patterns in 2015). Hence, the only difference is how community composition varies across the land uses for each taxonomic group.

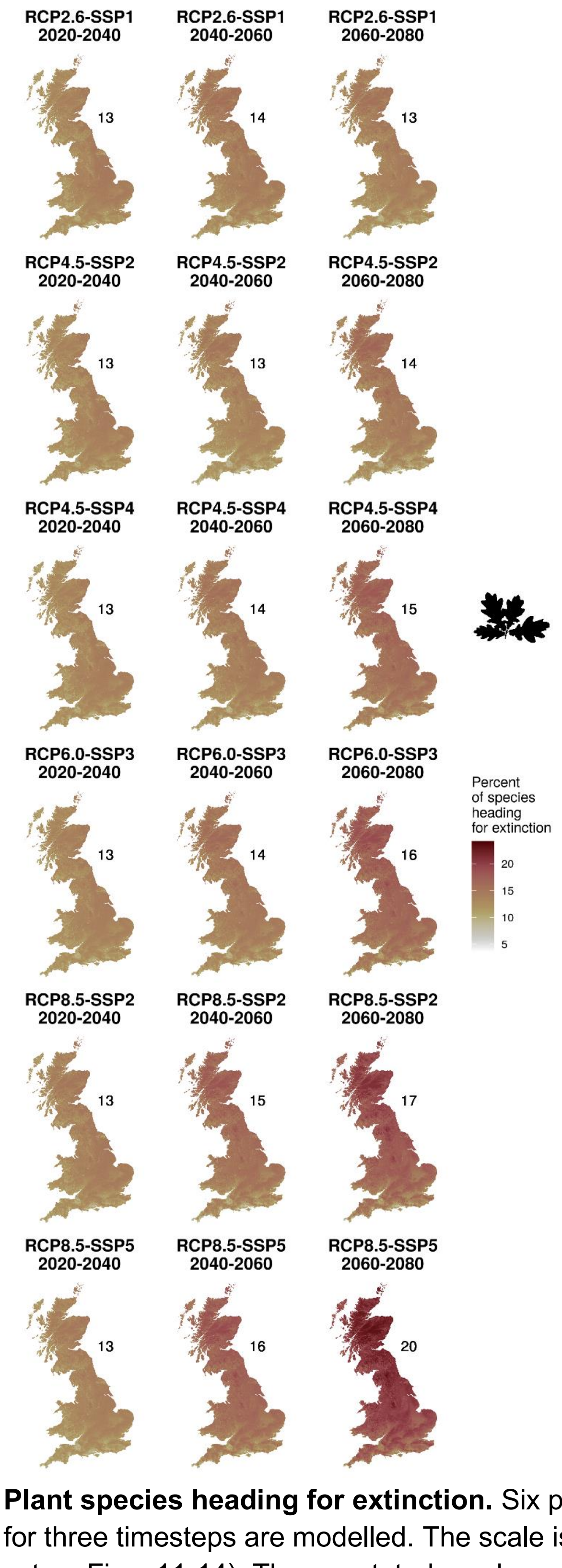

Supplementary Fig. 12: Plant species heading for extinction. Six plausible futures (Supplementary Table 1) for three timesteps are modelled. The scale is comparable across extinction plots (Supplementary Figs. 11-14). The annotated numbers represent the weighted geometric mean across grid cells.

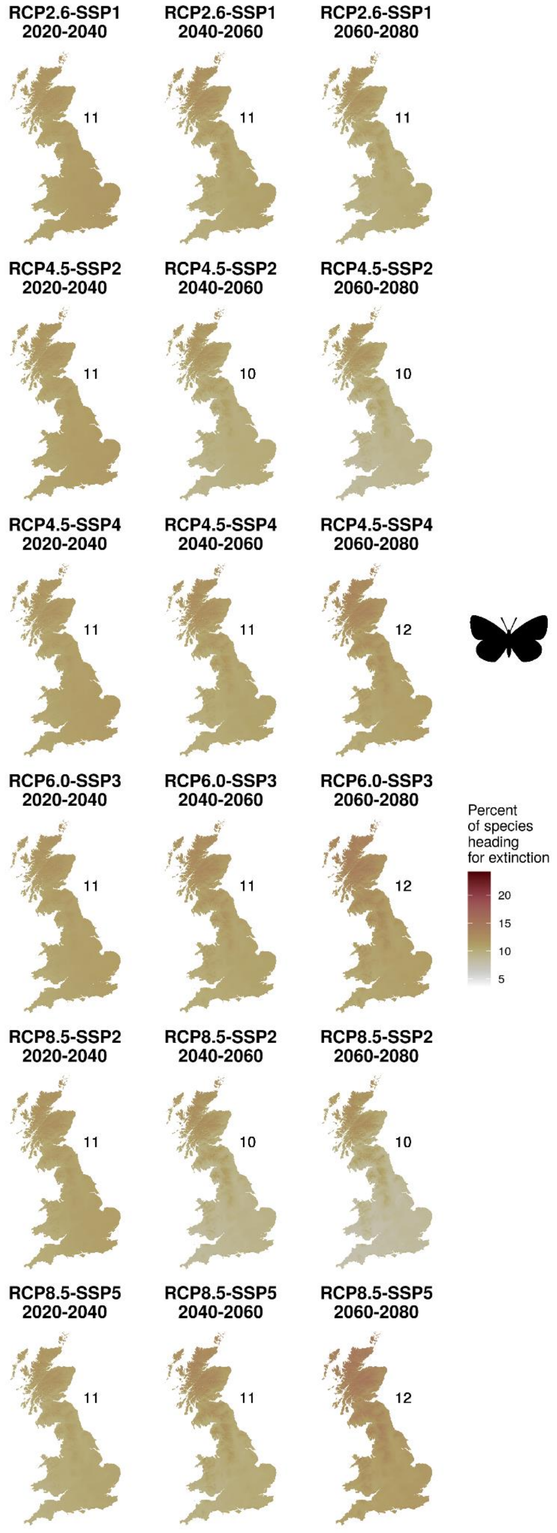

Supplementary Fig. 13: Butterfly species heading for extinction. Six plausible futures (Supplementary Table 1) for three timesteps are modelled. The scale is comparable across extinction plots (Supplementary Figs. 11-14). The annotated numbers represent the weighted geometric mean across grid cells.

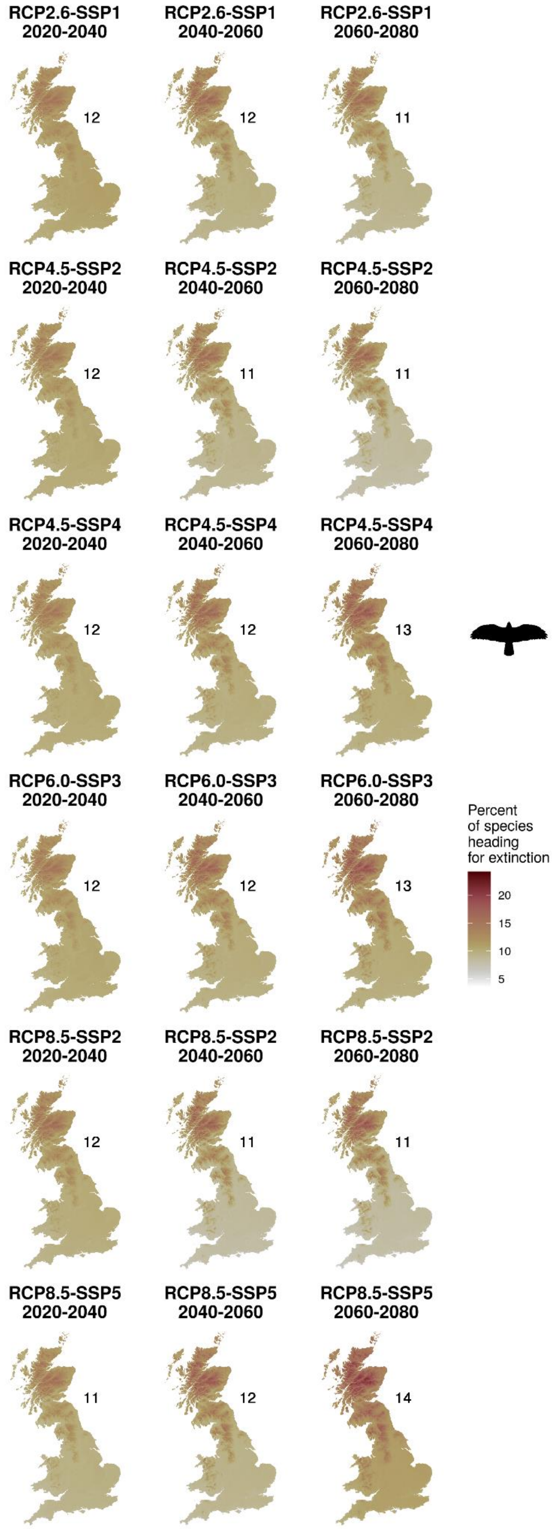

Supplementary Fig. 14: Bird species heading for extinction. Six plausible futures (Supplementary Table 1) for three timesteps are modelled. The scale is comparable across extinction plots (Supplementary Figs. 11-14). The annotated numbers represent the weighted geometric mean across grid cells.

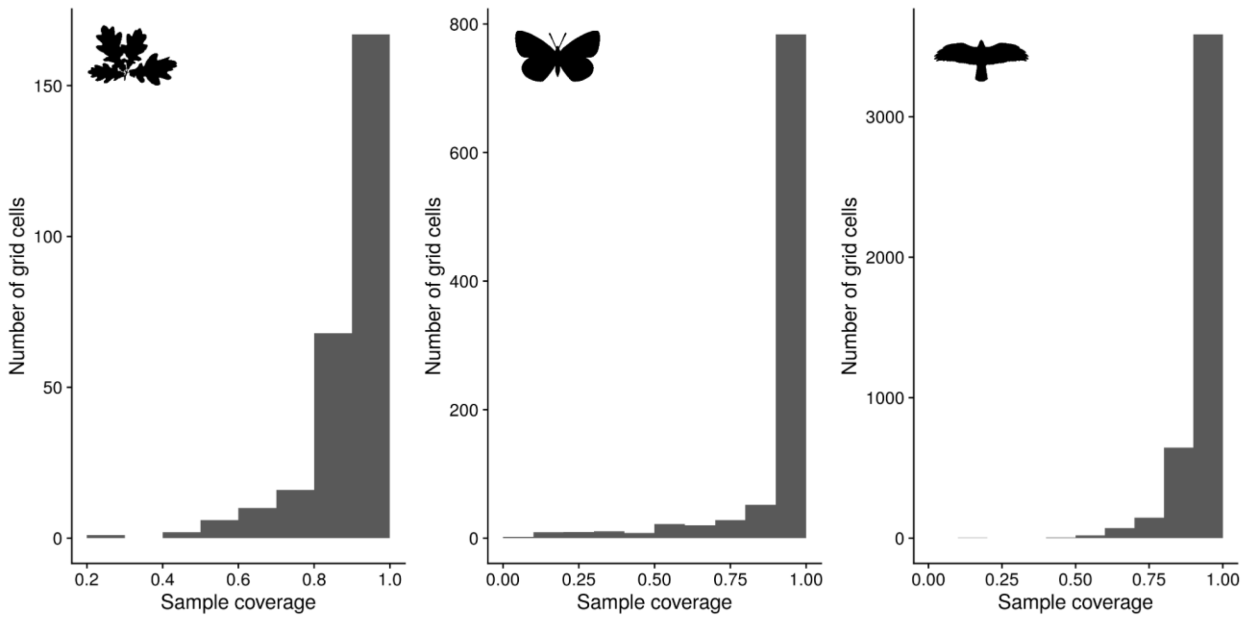

Supplementary Fig. 15: Histograms of estimated sample coverage across grid cells (count of grid cells) for plants, butterflies, and birds. Note different y axis limits.

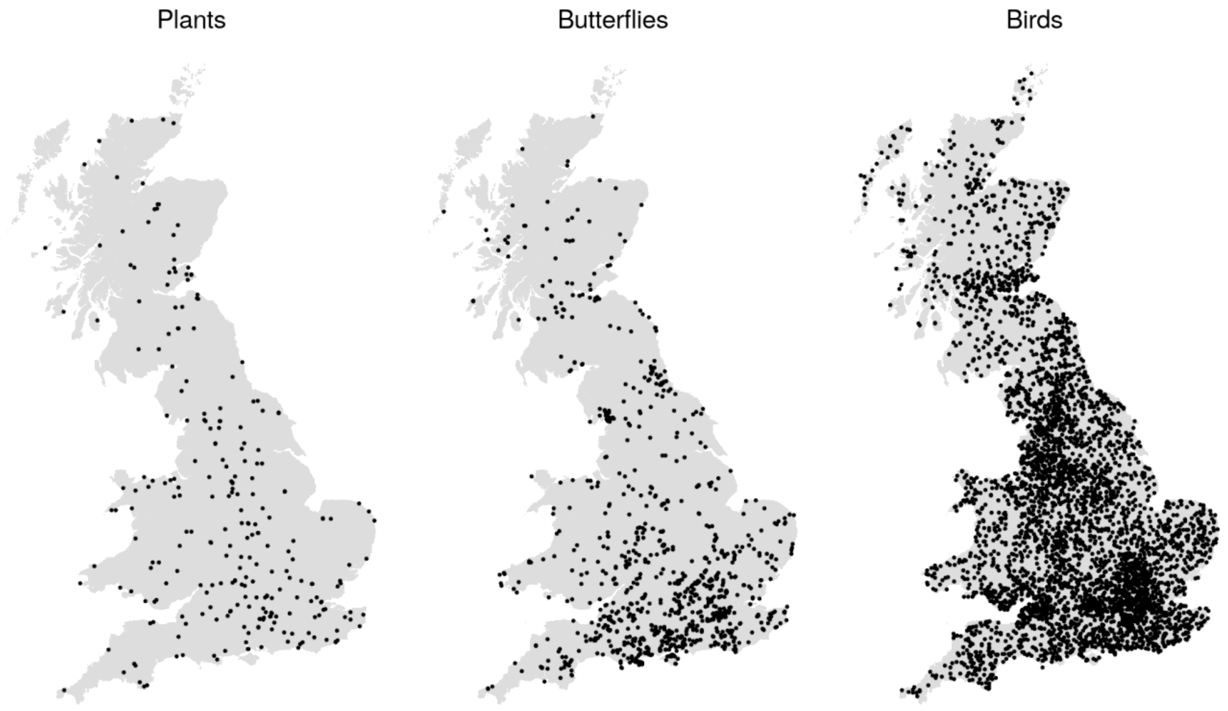

Supplementary Fig. 16: Locations of surveyed grid cells for plants, butterflies, and birds. 253 grid cells for plants, 926 grid cells for butterflies, 4,442 grid cells for birds.

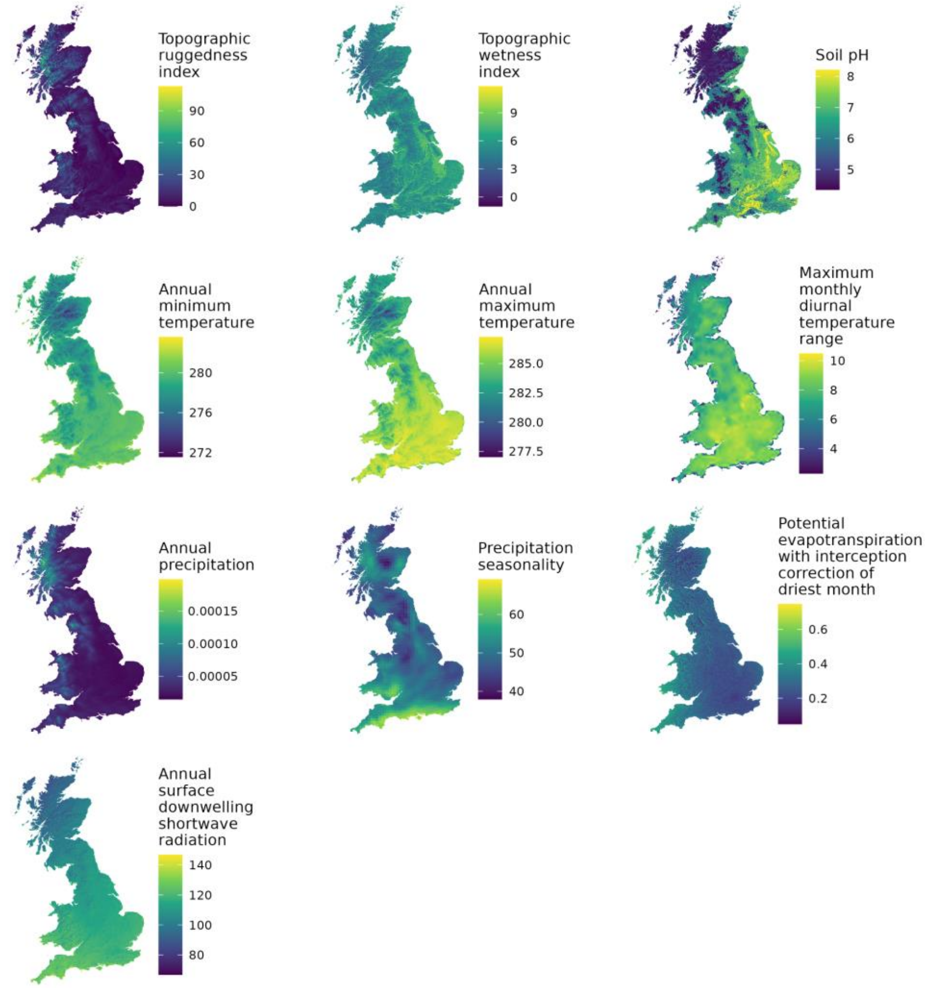

Supplementary Fig. 17: Baseline (1980-2010) environmental variables included in the baseline GDMs. Temperature (K), precipitation (kg m^{-2} s^{-1}), radiation (W m^{-2}).

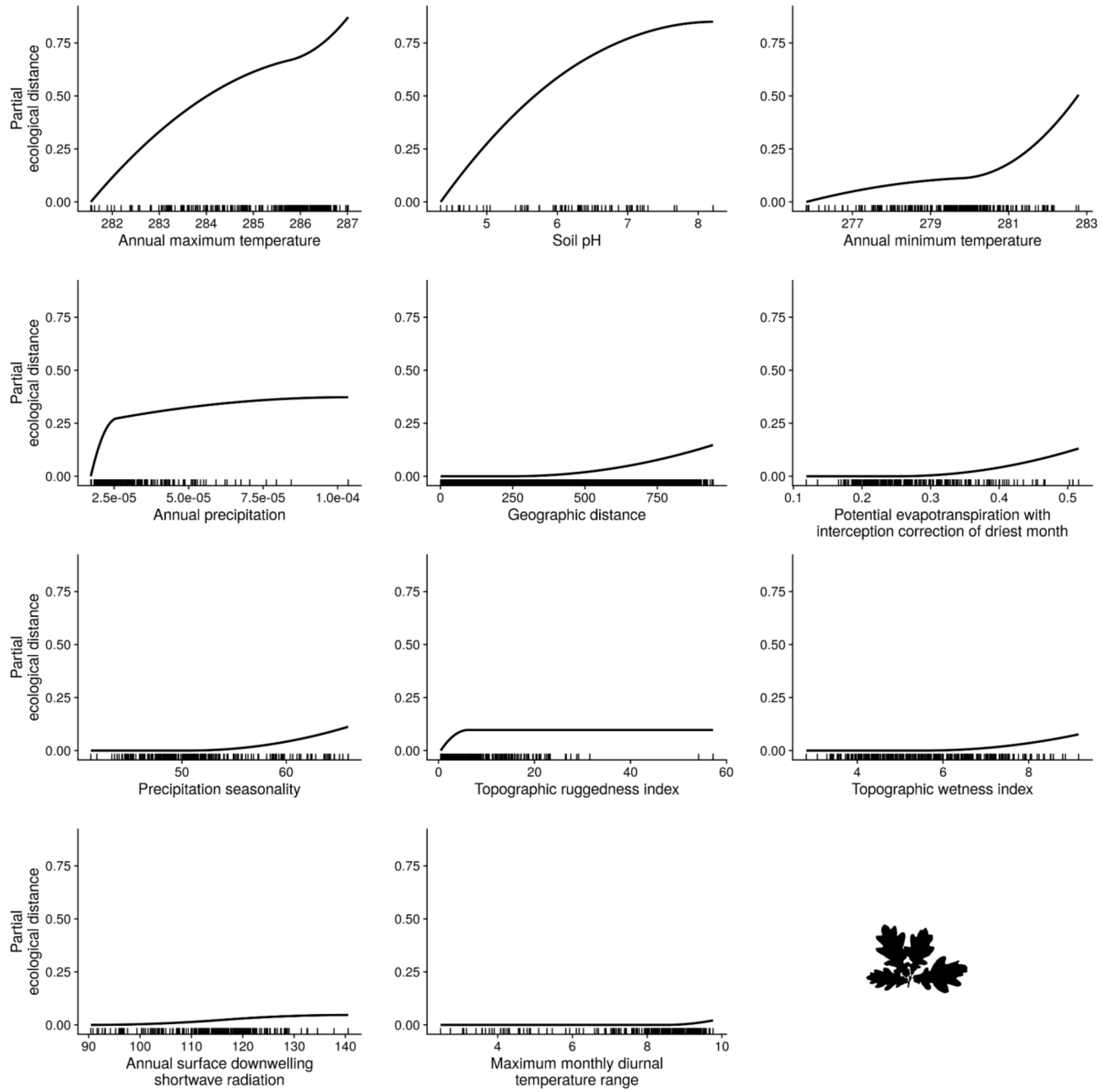

Supplementary Fig. 18: Generalised dissimilarity model-fitted I-splines linking plant compositional dissimilarity to environmental variables. Partial ecological distance indicates the local sensitivity of ecological distance, where ecological distance is related to dissimilarity (d_{ij}) via a link function; $-ln(1 - d_{ij})$. Maximum spline height indicates the total compositional change associated with the variables, while the slope indicates the rate of compositional change along the environmental gradient. Rugs show locations of observed data. Environmental variables are ordered based on their relative importance. Geographic distance (km), Temperature (K), precipitation (kg m^{-2} s^{-1}), radiation (W m^{-2}).

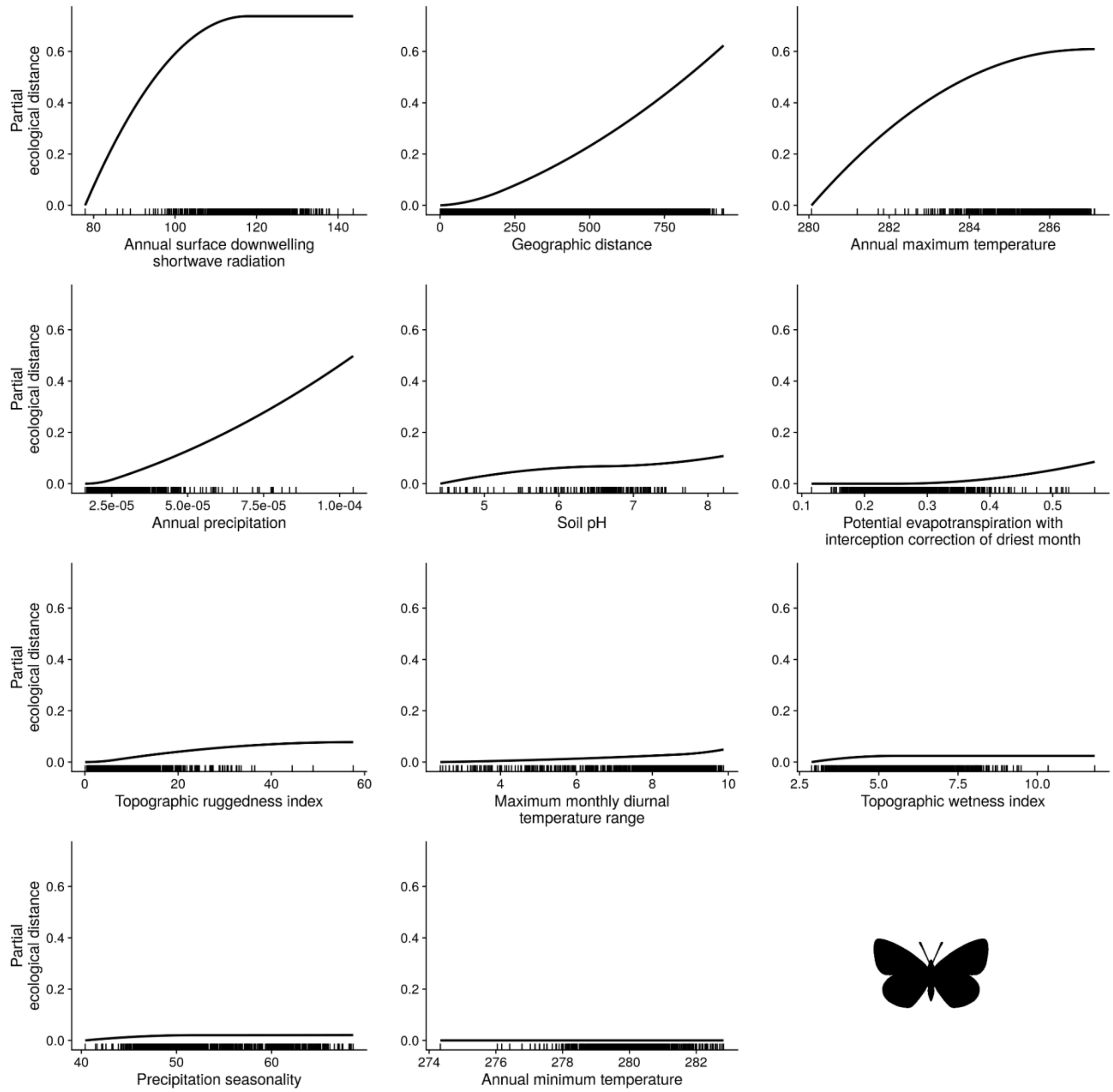

Supplementary Fig. 19: Generalised dissimilarity model-fitted I-splines linking butterfly compositional dissimilarity to environmental variables. Partial ecological distance indicates the local sensitivity of ecological distance, where ecological distance is related to dissimilarity (d_{ij}) via a link function; $-ln(1 - d_{ij})$. Maximum spline height indicates the total compositional change associated with the variables, while the slope indicates the rate of compositional change along the environmental gradient. Rugs show locations of observed data. Environmental variables are ordered based on their relative importance. Geographic distance (km), Temperature (K), precipitation (kg m^{-2} s^{-1}), radiation (W m^{-2}).

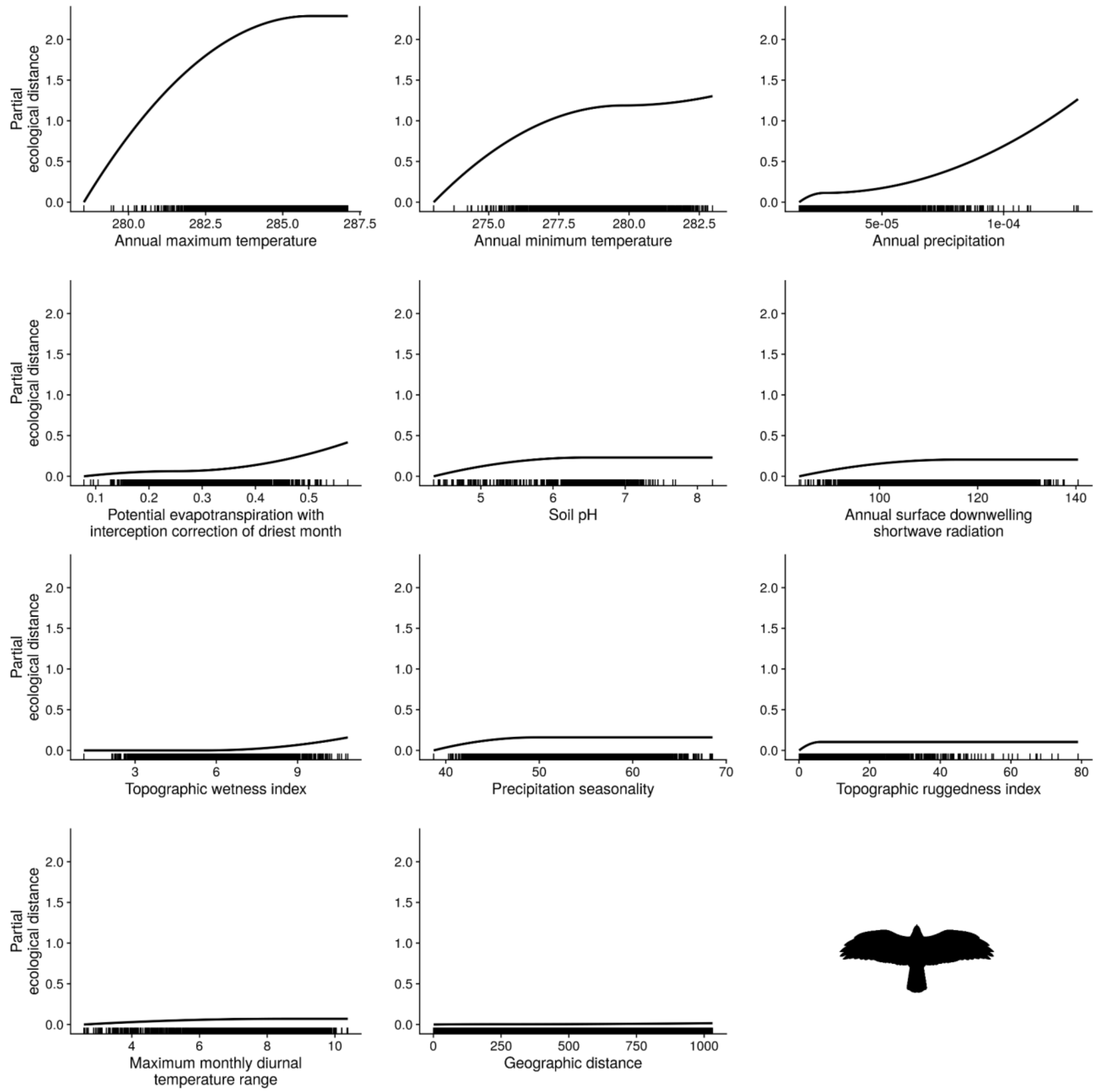

Supplementary Fig. 20: Generalised dissimilarity model-fitted I-splines linking bird compositional dissimilarity to environmental variables. Partial ecological distance indicates the local sensitivity of ecological distance, where ecological distance is related to dissimilarity (d_{ij}) via a link function; $-ln(1 - d_{ij})$. Maximum spline height indicates the total compositional change associated with the variables, while the slope indicates the rate of compositional change along the environmental gradient. Rugs show locations of observed data. Environmental variables are ordered based on their relative importance. Geographic distance (km), Temperature (K), precipitation (kg m^{-2} s^{-1}), radiation (W m^{-2}).

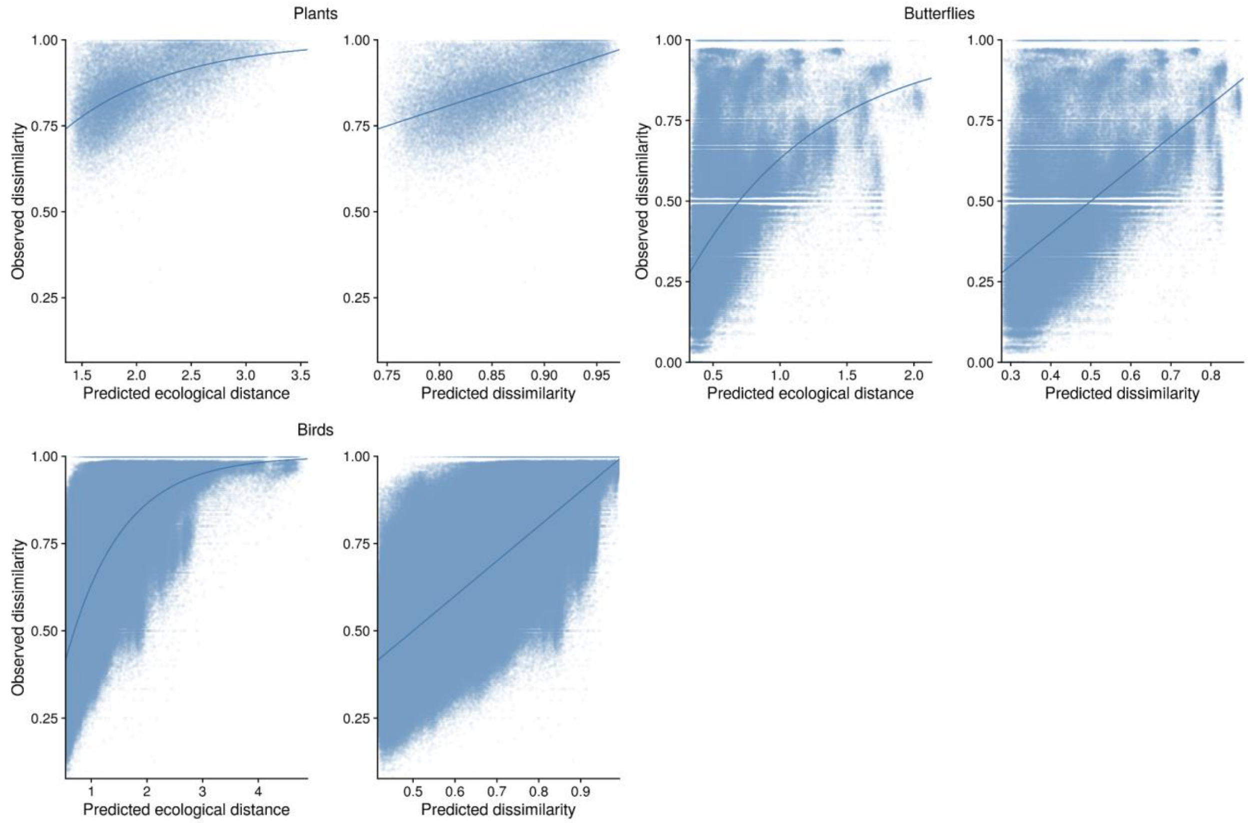

Supplementary Fig. 21: Baseline GDM fit for each taxonomic group. Shown for each group is (left) observed dissimilarity as a function of GDM-predicted ecological distance, with each site-pair represented as a point, and the line representing the GDM-predicted dissimilarity; and (right) observed dissimilarity as a function of GDM-predicted dissimilarity, with the line of equality provided. All models use 10 environmental predictors (Supplementary Fig. 17). Plants GDM applies all 31,878 site-pairs from 253 sites with compositional data, explaining 32% deviance in observed dissimilarities. Butterflies GDM: 428,275 site-pairs; 926 sites; 31% explained deviance. Birds GDM: 9,863,461 site-pairs; 4,442 sites; 56% explained deviance.

Supplementary Table 1: Plausible combinations of RCPs (Representative Concentration Pathways) and SSPs (Shared Socio-economic Pathways) are used to define plausible futures (grey).

	SSP1	SSP2	SSP3	SSP4	SSP5
RCP2.6					
RCP4.5					
RCP6.0					
RCP8.5					

Supplementary Table 2: Details on the six plausible futures investigated[1–5].

Plausible future	RCP	SSP	Key characteristics
RCP2.6-SSP1	RCP2.6 - low emissions, peak and then decline in radiative forcing. Global increase in temperature by 2081-2100 compared to 1850-1900 (mean ±1 standard deviation): 1.6 ± 0.4.	SSP1: Sustainability - shows the UK transitioning to a fully functional circular economy as society quickly becomes more egalitarian leading to healthier lifestyles, improved well-being, sustainable use of natural resources, and more stable and fair international relations. It represents a sustainable and co-operative society with a low carbon economy and high capacity to adapt to climate change.	Emphasis on **sustainable agricultural and forestry production** (increased mixed woodland and sustainable agriculture) and the delivery of multiple ecosystem services. Decreasing area of intensive agriculture. **Reduced meat demand**, shift away from livestock production, decrease in pastoral area. Semi-natural habitats in both the lowlands and uplands are effectively **restored and rewilded**.
RCP4.5-SSP2	RCP4.5 - low-moderate emissions, stabilisation. Global increase in temperature: 2.4 ± 0.5.	SSP2: Middle of the Road - a world in which strong public-private partnerships enable moderate economic growth but inequalities persist. It represents a highly regulated society that continues to rely on fossil fuels, but with gradual increases in renewable energy resulting in intermediate adaptation and mitigation challenges.	**Intensification** and increasing efficiency of agriculture. Large **increase in planted forest** area. Large **drop in meat and milk demand**, large decrease in intensive pasture area. Payment for Ecosystem Services schemes. Later in the scenario, reduced land use conflicts free up **more land for conservation**.
RCP4.5-SSP4	RCP4.5 - see above	SSP4: Inequality - shows how a society dominated by business and political elites may lead to increasing inequalities by curtailing welfare policies and excluding the majority of a disengaged population. The business and political elite facilitate low carbon economies but large differences in income across segments of UK society limits the adaptive capacity of the masses.	Large-scale **industrial agriculture**. Increasing arable extent and intensity, at expense of forest areas. Decrease in the relative demand for grass-fed livestock, **decline in pasture**. Demand for recreation by the rich elite, large areas of **abandonment and conservation management** emerge in **upland areas**. Loss of marginal land uses. The **easing of environmental protection** standards results in a general **decline in environmental health**, particularly in agricultural landscapes and urban areas.
RCP6.0-SSP3	RCP6.0 - moderate emissions, stabilisation. Global increase in	SSP3: Regional Rivalry - the dystopian scenario, shows how increasing social and economic barriers may trigger international tensions, nationalisation in key economic sectors, job losses and, eventually a highly fragmented	**Extensification** of production as inputs become unavailable, shortfalls in supply and increasing area with maximum possible intensity. Food production dominates land uses. Heterogeneous and frequent **changes in land use**, suboptimal exploitation of

	temperature: 2.8 ± 0.5.	society with the UK breaking apart. It represents a society where rivalry between regions and barriers to trade entrench reliance on fossil fuels and limit capacity to adapt to climate change.	available capitals. Divergence in land system trajectories between England, Wales and Scotland, with least intensive production methods being only feasible options in smaller nations. **Nature protection** quickly **declines** due to the lifting of environmental regulations and legal and social measures that restrict NGOs and crackdown on environmental activism groups. National Parks and other **protected areas** de facto **disappear** by the 2060s.
RCP8.5-SSP2	RCP8.5 - high emissions, rising radiative forcing. Global increase in temperature: 4.3 ± 0.7.	SSP2 - see above	**Similar to RCP4.5-SSP2**, but with **larger areas of forest** under RCP8.5 (demands for afforestation to sequester carbon and produce timber).
RCP8.5-SSP5	RCP8.5 - see above	SSP5: Fossil-fuelled Development - shows the UK transitioning to a highly individualistic society where the majority become wealthier through the exploitation of natural resources combined with high economic growth. It represents a technologically advanced world with a strong economy that is heavily dependent on fossil fuels, but with a high capacity to adapt to the impacts of climate change.	Massive **urban expansion** (large human population) and very high levels of **agricultural intensification**. **Increased pollution**. High demands for livestock products. **Protected areas removed**. **Land abandonment/recreation** in the **uplands and marginal areas**. Any protected areas on land that is profitable in the lowlands is quickly lost. **Weakened regulations** to protect the environment.

Supplementary Table 3: Translation between the target CRAFTY[3] land-use classes and the PREDICTS database[6] land-use classes.

CRAFTY land-use class	Harmonised land-use class	PREDICTS land-use class
Unmanaged	Reference	Primary vegetation minimal & light use; Mature and intermediate secondary vegetation minimal & light use
Agroforestry	Agroforestry (average across sustainable agriculture-extensive pastoral-productive woodland)	Annual crop minimal use; Perennial crop minimal use; Rangeland minimal and light use; Primary vegetation intense use; Mature and intermediate secondary vegetation intense use; Timber
Bioenergy	Intensive agriculture	Annual crop intense use; Perennial crop intense use
Extensive agriculture	Extensive agriculture	Annual crop light use; Perennial crop light use
Extensive pastoral	Extensive pastoral	Rangeland minimal and light use
Intensive agriculture fodder	Intensive agriculture	Annual crop intense use; Perennial crop intense use
Intensive agriculture food	Intensive agriculture	Annual crop intense use; Perennial crop intense use
Intensive pastoral	Intensive pastoral	Managed pasture intense and light use
Mixed woodland	Reference	Primary vegetation minimal & light use; Mature and intermediate secondary vegetation minimal & light use
Natural woodland conservation	Reference	Primary vegetation minimal & light use; Mature and intermediate secondary vegetation minimal & light use
Productive native broadleaf	Productive woodland	Primary vegetation intense use; Mature and intermediate secondary vegetation intense use; Timber
Productive native conifer	Productive woodland	Primary vegetation intense use; Mature and intermediate secondary vegetation intense use; Timber
Productive non-native broadleaf	Productive woodland	Primary vegetation intense use; Mature and intermediate secondary vegetation intense use; Timber

Productive non-native conifer	Productive woodland	Primary vegetation intense use; Mature and intermediate secondary vegetation intense use; Timber
Sustainable agriculture	Sustainable agriculture	Annual crop minimal use; Perennial crop minimal use
Very extensive pastoral	Extensive pastoral	Rangeland minimal and light use
Urban	Urban	Urban

Supplementary Table 4: Biodiversity Intactness Index (BII) coefficients representing the proportional native species total organism abundance and compositional similarity expected to be retained in each land-use class, relative to a pristine environment (Reference).

Harmonised land use class	BII coefficient	Number of sites in abundance model	Number of pairwise comparisons for compositional similarity
Reference	1.00	7,067	316,559 (Reference-Reference)
Productive woodland	0.80	2,944	69,378 (Reference-Productive woodland)
Extensive pastoral	0.80	1,198	26,380 (Reference-Extensive pastoral)
Agroforestry	0.73	Agroforestry is constructed from the mean of the coefficients for sustainable agriculture, extensive pastoral and productive woodland	
Intensive pastoral	0.63	4,561	33,808 (Reference-Intensive pastoral)
Hyper-intensive pastoral	2040 = 0.62<br>2050 = 0.58<br>2060 = 0.54<br>2070 = 0.50<br>2080 = 0.50	Extrapolated from intensive pastoral based on average intensity into the future (SSP5 only)	
Sustainable agriculture	0.59	585	4,471 (Reference-Sustainable agriculture)
Extensive agriculture	0.49	1,177	6,236 (Reference-Extensive agriculture)
Intensive agriculture	0.43	1,389	11,749 (Reference-Intensive agriculture)
Hyper-intensive agriculture	2040 = 0.43<br>2050 = 0.41<br>2060 = 0.40<br>2070 = 0.39<br>2080 = 0.39	Extrapolated from intensive agriculture based on average intensity into the future (SSP5 only)	
Urban	0.36	907	17,596 (Reference-Urban)

Supplementary References

1. van Vuuren, D. P. *et al.* The representative concentration pathways: an overview. *Clim. Change* **109**, 5 (2011).
2. Collins, M. *et al.* Chapter 12 - Long-term climate change: Projections, commitments and irreversibility. in (ed. IPCC) (Cambridge University Press, Cambridge, 2013).
3. Brown, C. *et al.* Agent-Based Modeling of Alternative Futures in the British Land Use System. *Earths Future* **10**, e2022EF002905 (2022).
4. Harmáčková, Z. V. *et al.* Improving regional applicability of the UK shared socioeconomic Pathways through iterative participatory co-design. *Clim. Risk Manag.* **37**, 100452 (2022).
5. UK-SCAPE. UK Shared Socioeconomic Pathways (UK-SSPs). *SPEED* https://uk-scape.ceh.ac.uk/our-science/projects/SPEED/shared-socioeconomic-pathways (2024).
6. Hudson, L. N. *et al.* The database of the PREDICTS (Projecting Responses of Ecological Diversity In Changing Terrestrial Systems) project. *Ecol. Evol.* **7**, 145–188 (2017).
