# Supplementary material for: Future scenarios for British biodiversity under climate and land-use change
Source: Nat Commun. 2026 Mar 31;17:2704. doi: 10.1038/s41467-026-70064-4 (PMC13039725; doi:10.1038/s41467-026-70064-4)
Supplement: Supplementary file 2 — Reporting Summary [file 41467_2026_70064_MOESM2_ESM.pdf]

nature portfolio

Corresponding author(s): Rob Cooke

Last updated by author(s): Jan 23, 2026

Reporting Summary

Nature Portfolio wishes to improve the reproducibility of the work that we publish. This form provides structure for consistency and transparency in reporting. For further information on Nature Portfolio policies, see our Editorial Policies and the Editorial Policy Checklist.

Statistics

For all statistical analyses, confirm that the following items are present in the figure legend, table legend, main text, or Methods section.

n/a	Confirmed	
☐	☒	The exact sample size (*n*) for each experimental group/condition, given as a discrete number and unit of measurement
☒	☐	A statement on whether measurements were taken from distinct samples or whether the same sample was measured repeatedly
☒	☐	The statistical test(s) used AND whether they are one- or two-sided<br>*Only common tests should be described solely by name; describe more complex techniques in the Methods section.*
☐	☒	A description of all covariates tested
☒	☐	A description of any assumptions or corrections, such as tests of normality and adjustment for multiple comparisons
☐	☒	A full description of the statistical parameters including central tendency (e.g. means) or other basic estimates (e.g. regression coefficient) AND variation (e.g. standard deviation) or associated estimates of uncertainty (e.g. confidence intervals)
☒	☐	For null hypothesis testing, the test statistic (e.g. *F*, *t*, *r*) with confidence intervals, effect sizes, degrees of freedom and *P* value noted<br>*Give P values as exact values whenever suitable.*
☒	☐	For Bayesian analysis, information on the choice of priors and Markov chain Monte Carlo settings
☒	☐	For hierarchical and complex designs, identification of the appropriate level for tests and full reporting of outcomes
☐	☒	Estimates of effect sizes (e.g. Cohen's *d*, Pearson's *r*), indicating how they were calculated

Our web collection on statistics for biologists contains articles on many of the points above.

Software and code

Policy information about availability of computer code

Data collection	No software was used for data collection
Data analysis	The primary code for data analysis was written in R version 4.4.0. See Zenodo for R code summarising the major analytical steps: https://doi.org/10.5281/zenodo.14834251. R packages used to perform the analyses are listed in the renv.lock file. Specifically, we used multiple R packages (and their dependencies) for data preparation, analysis, and visualization, including blme 1.0-5, BRCmap 0.11.0.1, corrplot 0.95, cowplot 1.1.3, dplyr 1.1.4, gdm 1.6.0-4, gghalves 0.1.4, ggplot2 3.5.1, gower 1.0.1, gstat 2.1-1, iNEXT 3.0.1, janitor 2.2.0, lme4 1.1-31, ncdf4 1.22, pbmcapply 1.5.1, raster 3.6-30, rphylopic 1.5.01, scico 1.5.0, sf 1.0-16, sp 2.1-4, terra 1.8-5, tibble 3.2.1, tidyr 1.3.1.

For manuscripts utilizing custom algorithms or software that are central to the research but not yet described in published literature, software must be made available to editors and reviewers. We strongly encourage code deposition in a community repository (e.g. GitHub). See the Nature Portfolio guidelines for submitting code & software for further information.

Data

Policy information about availability of data

All manuscripts must include a data availability statement. This statement should provide the following information, where applicable:

- Accession codes, unique identifiers, or web links for publicly available datasets
- A description of any restrictions on data availability
- For clinical datasets or third party data, please ensure that the statement adheres to our policy

All prepared and processed data are available at https://doi.org/10.5281/zenodo.14834251.

Raw data are available from the original sources. Structured record data for plants: https://doi.org/10.5285/e742c94f-82a4-43e7-af14-36b131afe81b. Structured record data for butterflies: https://doi.org/10.5285/b77561d8-20ee-45ad-9221-7a8885d5ac8e. Structured record data for birds can be requested: https://www.bto.org/our-science/data/data-request-system. Topographic ruggedness index data: https://doi.org/10.1594/PANGAEA.867115. Topographic wetness index data: https://doi.org/10.5285/6b0c4358-2bf3-4924-aa8f-793d468b92be. Modelled estimates of soil pH: https://doi.org/10.5285/4b0e364d-61e6-48fb-8973-5eb18fb454cd. Climatic data: https://dx.doi.org/10.5285/8194b416cbee482b89e0dfbe17c5786c. Land use projections: https://doi.org/10.5285/f9ab3051-4f85-415f-b691-371ff8e951f2. Biodiversity samples from the PREDICTS database: https://data.nhm.ac.uk/dataset/the-2016-release-of-the-predicts-database and https://data.nhm.ac.uk/dataset/release-of-data-added-to-the-predicts-database-november-2022. WorldClim data: https://worldclim.org/. Net primary productivity data: https://doi.org/10.5067/MODIS/MOD17A3HGF.061.

Research involving human participants, their data, or biological material

Policy information about studies with human participants or human data. See also policy information about sex, gender (identity/presentation), and sexual orientation and race, ethnicity and racism.

Reporting on sex and gender	NA
Reporting on race, ethnicity, or other socially relevant groupings	NA
Population characteristics	NA
Recruitment	NA
Ethics oversight	NA

Note that full information on the approval of the study protocol must also be provided in the manuscript.

Field-specific reporting

Please select the one below that is the best fit for your research. If you are not sure, read the appropriate sections before making your selection.

☐ Life sciences ☐ Behavioural & social sciences ☒ Ecological, evolutionary & environmental sciences

For a reference copy of the document with all sections, see nature.com/documents/nr-reporting-summary-flat.pdf

Ecological, evolutionary & environmental sciences study design

All studies must disclose on these points even when the disclosure is negative.

Study description	Using available data we modelled and projected the magnitude and spatial distribution of biodiversity change under a range of climate and land-use scenarios across Great Britain up to the year 2080.
Research sample	1002 plant species, 56 butterfly species, and 219 bird species across Great Britain. Specifically, we prepared biological data for 1002 plant species (~72% of British native plant species) between 2015 and 2021 across 253 grid cells, for 56 butterfly species (~97% of British native butterfly species) between 2001 and 2010 across 926 grid cells, and for 245 breeding bird species (~96% of British native breeding bird species) between 2002 and 2010 across 4,445 grid cells. The environmental data cover Great Britain (229,752 grid cells).
Sampling strategy	All species with available structured monitoring data were included. No sample size calculation was performed. Data covered a sizeable proportion of British native plant species (~72%), butterfly species (~97%) and breeding bird species (~96%).
Data collection	Data were compiled from multiple sources and are cited in the manuscript. The data included structured biological data for plants, butterflies and birds. As well as environmental data, and future climate and land use data.
Timing and spatial scale	The published biological data for plants was sampled between 2015 and 2021, for butterflies between 2001 and 2010, and for birds between 2002 and 2010. Spatially, the biological data covers parts of Great Britain at ~1km resolution, 253 grid cells for plants, 926 grid cells for butterflies, and 4,445 grid cells for breeding birds.
Data exclusions	We excluded biological data from grid cells where sample coverage could not be reliably estimated (cells with less than two sampled years).
Reproducibility	No experimental procedures were undertaken. Analytical reproducibility is underpinned by the R analysis code available on Zenodo https://doi.org/10.5281/zenodo.14834251 (DOI: 10.5281/zenodo.14834251).
Randomization	No experiments were conducted for this study.
Blinding	No experiments were conducted for this study.

Did the study involve field work? ☐ Yes ☒ No

Reporting for specific materials, systems and methods

We require information from authors about some types of materials, experimental systems and methods used in many studies. Here, indicate whether each material, system or method listed is relevant to your study. If you are not sure if a list item applies to your research, read the appropriate section before selecting a response.

Materials & experimental systems

n/a	Involved in the study
☒	☐ Antibodies
☒	☐ Eukaryotic cell lines
☒	☐ Palaeontology and archaeology
☒	☐ Animals and other organisms
☒	☐ Clinical data
☒	☐ Dual use research of concern
☒	☐ Plants

Methods

n/a	Involved in the study
☒	☐ ChIP-seq
☒	☐ Flow cytometry
☒	☐ MRI-based neuroimaging

Plants

Seed stocks	NA
Novel plant genotypes	NA
Authentication	NA
